# Loss of PBAF promotes expansion and effector differentiation of CD8^+^ T cells during chronic viral infection and cancer

**DOI:** 10.1016/j.celrep.2023.112649

**Published:** 2023-06-16

**Authors:** Arjun Kharel, Jian Shen, Ryan Brown, Yao Chen, Christine Nguyen, Donia Alson, Theresa Bluemn, Jie Fan, Kexin Gai, Bin Zhang, Matthew Kudek, Nan Zhu, Weiguo Cui

**Affiliations:** 1 Department of Pathology, Feinberg School of Medicine, Northwestern University, Chicago, IL, USA; 2 Department of Medicine/Hematology and Oncology, Feinberg School of Medicine, Northwestern University, Chicago, IL, USA; 3 Department of Microbiology-Immunology, Northwestern University, Chicago, IL, USA; 4 Blood Research Institute, Versiti, Milwaukee, WI, USA; 5 Department of Microbiology and Immunology, Medical College of Wisconsin, Milwaukee, WI, USA; 6 Department of Cell Biology, Neurobiology and Anatomy, Medical College of Wisconsin, Milwaukee, WI, USA; 7 Shanghai Institute of Immunology, Department of Immunology and Microbiology, Shanghai Jiao Tong University School of Medicine, Shanghai, China; 8 These author contributed equally; 9 Lead contact

## Abstract

During chronic viral infection and cancer, it has been established that a subset of progenitor CD8^+^ T cells continuously gives rise to terminally exhausted cells and cytotoxic effector cells. Although multiple transcriptional programs governing the bifurcated differentiation trajectories have been previously studied, little is known about the chromatin structure changes regulating CD8^+^ T cell-fate decision. In this study, we demonstrate that the chromatin remodeling complex PBAF restrains expansion and promotes exhaustion of CD8^+^ T cells during chronic viral infection and cancer. Mechanistically, transcriptomic and epigenomic analyses reveal the role of PBAF in maintaining chromatin accessibility of multiple genetic pathways and transcriptional programs to restrain proliferation and promote T cell exhaustion. Harnessing this knowledge, we demonstrate that perturbation of PBAF complex constrained exhaustion and promoted expansion of tumor-specific CD8^+^ T cells resulting in antitumor immunity in a preclinical melanoma model, implicating PBAF as an attractive target for cancer immunotherapeutic.

## In brief

During chronic viral infection, progenitor CD8^+^ T cells undergo bifurcated differentiation toward effector and exhausted lineages, but the chromatin structure changes orchestrating this transition remain unclear. Kharel et al. demonstrate that the chromatin remodeler PBAF regulates exhaustion-associated chromatin and transcriptional changes to restrict expansion and promote exhaustion of CD8^+^ T cells.

## Graphical Abstract

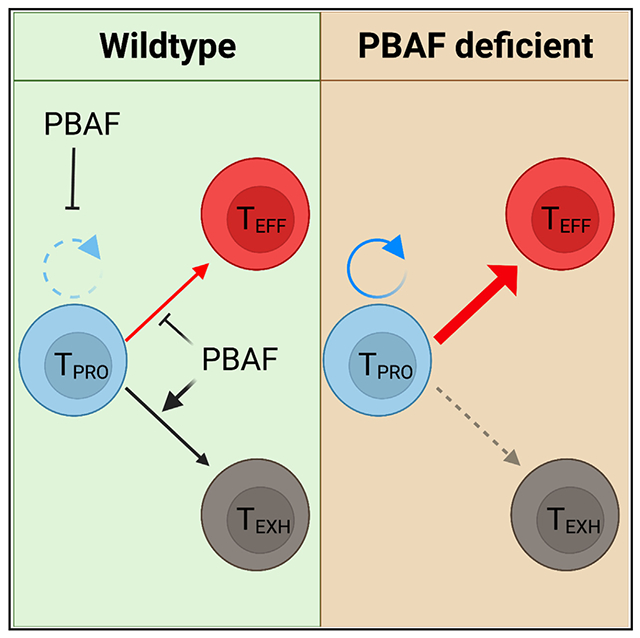

## INTRODUCTION

CD8^+^ T cells responding to chronic viral infection and cancer gradually become dysfunctional, commonly known as T cell exhaustion.^1–3^ Exhausted CD8^+^ T cells are characterized by loss of cytokine and effector molecule production, high expression of coinhibitory receptors (such as PD-1, LAG-3, and 2B4), altered cellular metabolism, and impaired proliferative potential.^4,5^ Recently, a growing body of evidence indicates that the previously known exhausted CD8^+^ T cell pool is quite heterogeneous and consists of at least three major phenotypically and functionally distinct subsets.^6–16^ A TCF-1^hi^ progenitor (T_PRO_) subset functions as a population of self-renewing resource cells that continuously replenish the pool of terminally exhausted T cells (T_EXH_)^8,10^ and a CX_3_CR1^+^ effector subset (T_EFF_) with enhanced killing ability.^9,11,15,16^

It has become increasingly clear that the heterogeneity of CD8^+^ T cells and their bifurcated cellular differentiation is orchestrated by distinct transcriptional and epigenetic programs.^17,18^ The transcription factors (TFs) TCF-1, BACH2, and MyB are crucial to maintaining the stem cell-like features of progenitor CD8^+^ T cells.^10,19–21^ Likewise, several TFs drive the gene expression program of T_EXH_, including NFAT, NR4A proteins, EOMES, IRF4, IRF7, BLIMP-1, and TOX.^10,22–33^ More recently, we and others have shown that BATF, T-bet, and Zeb2 are critically required for T_EFF_ subset differentiation and function.^16,34,35^ Despite of these advances, how CD8^+^ T cell differentiation is epigenetically regulated remains poorly defined. Recent studies have characterized the chromatin accessibility as well as active and repressive histone marks in lymphocytic choriomeningitis virus (LCMV)-specific CD8^+^ T cells and revealed that the differentiation of heterogeneous CD8^+^ T cell subsets was driven by distinct gene regulatory networks and enhancer repertories.^34–36^ This raises an important question of what key chromatin remodeling events are required for cell-fate decisions in forming T_EFF_ versus T_EXH_ subsets.

The SWI/SNF complex is a multiunit chromatin remodeling complex that utilizes the energy of ATP hydrolysis to modulate chromatin architecture and regulate gene expression by sliding and displacing nucleosomes.^37–40^ The SWI/SNF complex exists in three distinct assemblies: canonical BAF (cBAF), polybromo-associated BAF (PBAF), and the non-cBAF (ncBAF) complex.^37,38^ These complexes are critical in T cell development, activation, proliferation, and differentiation.^41–43^ The chromatin remodeling activity of cBAF has been found to regulate CD4^+^ selection and enhance Th1 and Th17 differentiation.^41,44,45^ In addition, ncBAF is shown to promote the expression of Foxp3 in regulatory T (T_reg_) cells.^46^ More recently, the role of cBAF in CD8^+^ T cell differentiation and function has been unraveled by CRISPR screens.^47,48^ The deletion of Arid1a, a cBAF component, led to memory cell properties in CD8^+^ T cells that can be exploited to improve the efficacy of cancer immunotherapy.^47,48^ However, the detailed mechanisms by which BAF complexes regulate cell-fate decisions between effector and exhausted CD8^+^ T cells remain unknown.

In this study, we investigated the role of the PBAF complex in CD8^+^ T cell expansion and differentiation during chronic LCMV infection and cancer. We demonstrate that PBAF restricts expansion and promotes T_PRO_-to-T_EXH_ transition during chronic viral infection and cancer. Mechanistically, transcriptomic and epigenomic profling revealed that the altered gene expression and epigenetic landscape caused by the loss of PBAF favored proliferation, survival, effector cell differentiation, and limited T cell exhaustion. Single-cell multiomics further demonstrated the underlying chromatin structure changes accounting for the increased proliferative potential and preferential cell-fate commitment of the T_PRO_ cells toward T_EFF_ in the absence of the PBAF complex. Lastly, harnessing the role of PBAF in regulating expansion and effector cell-fate decision, we demonstrated increased antitumor function of PBAF-defcient CD8^+^ T cells in a preclinical melanoma model.

## RESULTS

### Loss of Arid2 promotes expansion and T_PRO_-to-T_EFF_ differentiation transition of LCMV-specific CD8^+^ T cells

To determine if PBAF deficiency affected CD8^+^ T cell clonal expansion and differentiation during chronic viral infection, we infected *Arid2*^*fl/fl*^ VavCre^+^ mice with LCMV Clone13 (Cl13) and assessed CD8^+^ T cell responses on day 21 post-infection (p.i.). Arid2 is a subunit unique to the PBAF complex whose genomic deletion results in defective assembly of the PBAF complex.^49^ CD8^+^ T cells specific to the LCMV GP_33-41_ epitope were measured by H-2D^b^ tetramer. In the spleen, *Arid2*^*fl/fl*^ VavCre^+^ mice had a significantly higher frequency and number of GP_33-41_ tetramer^+^ CD8^+^ T cells compared with their counterparts in *Arid2*^+/+^VavCre^+^ control mice (Figures S1A–S1C). Notably, Arid2-defcient mice exhibited a significant increase in the frequency of CX_3_CR_1_^+^ (T_EFF_) LCMV-specific CD8^+^ T cells, which was accompanied by a decrease in the frequency of Ly108^+^ (T_PRO_) and CX_3_CR1^−^Ly108^−^ (T_EXH_) in the spleen (Figure S1D), compared with their control counterparts, suggesting a potential role of the PBAF complex in orchestrating the expansion and subset distribution of CD8^+^ T cells during chronic viral infection.

**Figure 1.**
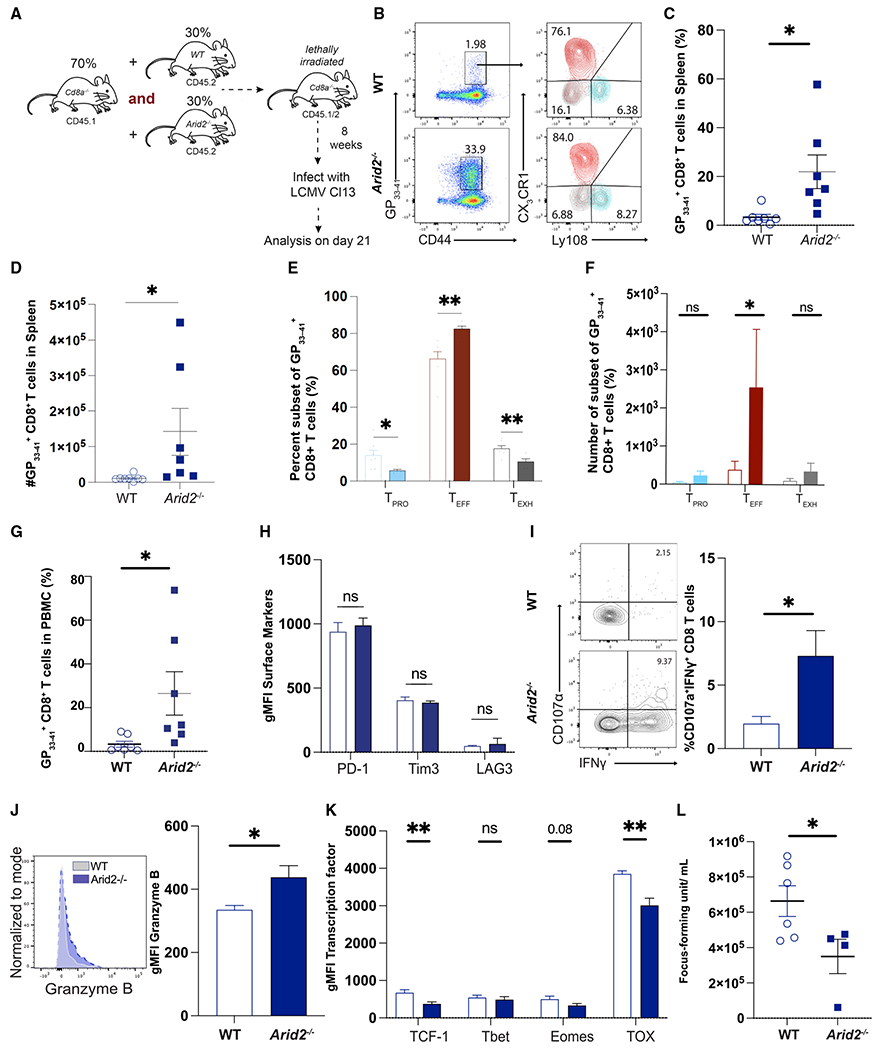
PBAF restricts expansion and promotes exhaustion of LCMV-specific CD8^+^ cells (A) Mixed bone marrow (MBM) chimeric mice reconstituted with CD45.1 *CD8α*^−/−^ and wild-type CD45.2 or *Arid2*^−/−^ CD45.2 were infected with LCMV clone 13 (Cl13) and analyzed on day 21 p.i. (B) Representative flow plots showing the frequency of GP33^+^ splenic CD8^+^ T cells in MBM chimera mice. (C–G) Summary data showing the proportion, subset distribution, and total number of GP33^+^ splenic CD8^+^ T cells in MBM chimera mice. (H) Summary data displaying the per cell expression (geometric mean fluorescence intensity [gMFI]) of PD-1, LAG3, and TIM3 in GP33^+^ splenic CD8^+^ T cells. (I) Representative flow plots and summary data showing the proportion of IFN-γ^+^CD8^+^ T cells. (J) Representative flow plots and summary data showing gMFI of granzyme B in GP33^+^ splenic CD8^+^ T cells. (K) Summary data showing the gMFI of TCF1, T-bet, Eomes, and TOX in GP33^+^ splenic CD8^+^ T cells. (L) Scatterplot displaying viral load in the sera. Summary data (mean ± SEM) are pooled from 2 experiments with at least 3 mice/group/experiment. Data are representative of two independent experiments. *p < 0.05, **p < 0.01, ***p < 0.0001.

**Figure 2.**
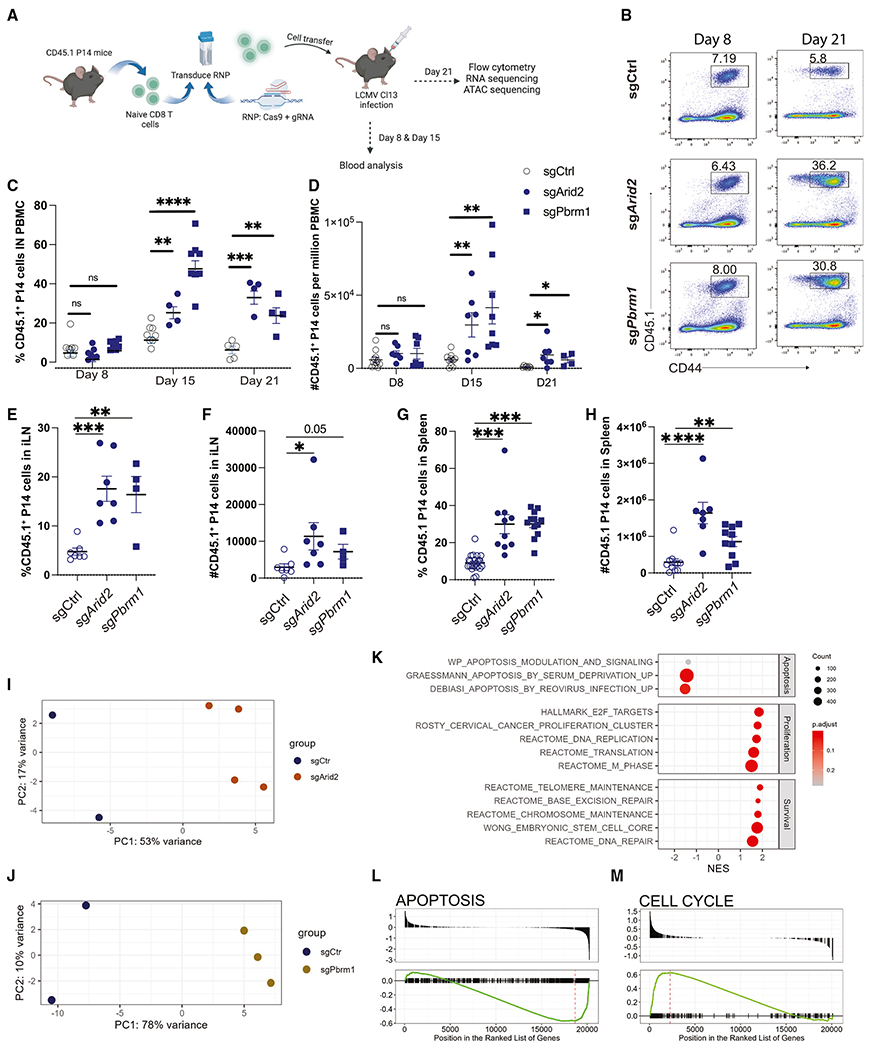
PBAF regulates expansion of virus-specific CD8^+^ T cells during the late phase of infection (A) CD45.1^+^P14^+^CD8^+^ T cells transduced with sgCtrl and *Arid2* or *Pbrm1* guide RNA were adoptively transferred into CD45.2^+^ C57BL/6 mice that were subsequently infected with LCMV Cl13. (B) Representative flow plots showing the frequency of control and *Arid2*- or *Pbrm1*-deleted CD8^+^ T cells on days 8 and 21. (C and D) Proportion and frequency of CD45.1^+^ cells in PBMCs on days 8, 15, and 21. (E–H) Frequency and absolute numbers of CD45.1^+^ cells in lymph nodes and spleen on day 21 p.i. (I) PCA plot of bulk RNA-seq of sgCtrl and sgArid2. (J) PCA plot of bulk RNA-seq of sgCtrl and sgPbrm1. (K) GSEA showing pathways significantly up- or downregulated in chronically infected Pbrm1-deficient P14 T cells. (L and M) Enrichment plots showing representative gene sets identified in the GSEA of Pbrm1-deficient P14 T cells. Summary data (mean ± SEM) in (C), (D), (G), and (H) are pooled from at least 2 independent experiments with at least 3 mice/group per experiment. (E) and (F) are from one independent experiment with at least 4 mice/group. Data in (B)–(D) are representative of three independent experiments. (I–M) Four replicates were included in each condition. *p < 0.05, **p < 0.01, ***p < 0.0001. Illustration created with BioRender.com.

In the *Arid2*^*fl/fl*^ VavCre^+^ mice, Cre recombinase gene expression is under the control of *Vav1* promoter that is constitutively active in hematopoietic cells throughout their lifespan.^50^ Although phenotypically, the differences in hematopoietic compartments between *Arid2*-defcient and wild-type groups were largely unchanged (Figures S1E–S1G), we sought to test the intrinsic role of the PBAF complex in CD8^+^ T cells during chronic infection. To this end, we generated mixed bone marrow (MBM) chimeric mice that restricted Arid2 deletion in CD8^+^ T cells (Figures 1A and S2A–S2C). We infected these chimeric mice with LCMV Cl13 and examined the virus-specific CD8^+^ T cell responses. On day 21 p.i., deletion of Arid2 resulted in a significantly higher frequency and number of GP_33-41_ tetramer^+^ CD8^+^ cells in the spleen (Figures 1B–1D). This increased proliferative burst was also observed in blood (Figure 2G). Importantly, loss of Arid2 in CD8^+^ T cells resulted in a significant increase in the frequency of the T_EFF_ subset, which was accompanied by a significant reduction in both T_EXH_ and T_PRO_ subsets (Figure 1E). Consistent with the increased clonal expansion, loss of Arid2 resulted in an increased number of virus-specific CD8^+^ T cells in all three subsets (Figure 1F). Interestingly, despite the increase in absolute number and frequency of the T_EFF_ subset, the expression levels of key inhibitory receptors such as PD-1, TIM-3, and LAG3 remained largely unchanged (Figure 1H). However, PBAF deficiency rendered a modest increase in effector function. GP_33–41_ peptide stimulation *ex vivo* showed a slightly higher frequency of interferon γ (IFNγ)^+^ CD8^+^T cells from *Arid2*^−/−^ mice than their counterparts (Figure 1I). Additionally, Arid2-deficient CD8^+^ T cells also exhibited a modest increase in granzyme B expression relative to their wild-type controls (Figure 1J). The sheer increase of virus-specific T_EFF_ CD8^+^ T cells in *Arid2*^−/−^ mice resulted in higher viral control (Figure 1L). Lastly, Arid2-defcient CD8^+^ T cells had significantly reduced expression of the TFs TCF-1 and TOX (Figure 1K), whereas no change in the expression of the TFs T-bet and Eomes was observed. Subset-specific analysis of TFs demonstrated a reduction in the expression of TCF-1 and TOX in all three major subsets following the loss of PBAF (Figures S2D–S2E). Collectively, these results demonstrate that Arid2 normally restrains clonal expansion and promotes T_PRO_-to-T_EXH_ differentiation of virus-specific CD8^+^ T cells during chronic infection.

### PBAF restricts the late-phase expansion of virus-specific CD8^+^ T cells

To determine if the phenotypic changes in Arid2-deficient CD8^+^ T cells are truly PBAF dependent, we decided to include Pbrm1, another subunit unique to the PBAF complex, in the investigation. To do this, we used a direct CRISPR-Cas9 delivery method by electroporation of the Cas9-gRNA ribonucleoprotein complex^51^ into naive CD8^+^ P14 cells that express a T cell receptor (TCR) transgene specific for the LCMV GP_33-41_ epitope to delete *Arid2* and *Pbrm1*. The validation of *Arid2* and *Pbrm1* deletion was performed by tracking of insertions or deletions (indels) by decomposition (TIDE) assay (Figures S3A–S3C) and western blot (Figure S3D), respectively. Next, we transferred the sg*Arid2*, sg*Pbrm1*, or scrambled-control gRNA (sgCtrl) electroporated P14 CD8^+^ T cells into three separate groups of naive congenic mice and subsequently infected them with LCMV Cl13 (Figure 2A). To demonstrate the role of PBAF in the clonal expansion of virus-specific CD8^+^ T cells, we examined the frequency of P14 cells in peripheral blood mononuclear cells (PBMCs) on days 8, 15, and 21 p.i. Although a similar clonal expansion was observed on day 8 p.i., the frequency and number of both *Arid2*- and *Pbrm1*-deleted P14 CD8^+^ T cells were significantly higher than their wild-type counterparts after the establishment of chronic exhaustion^31^ (days 15 and 21 p.i.) (Figures 2B–2D). Consistently, the frequency and numbers of PBAF-deficient P14T cells in spleen and inguinal lymph nodes (iLNs) were significantly increased compared with the wild-type control P14 T cells on day 21 p.i. (Figures 2E–2H).

To dissect the possible mechanisms by which the PBAF complex regulates CD8^+^ T cell proliferation during the late phase of chronic LCMV infection, we performed bulk RNA sequencing (RNA-seq) on *Pbrm1*- and *Arid2*-deleted P14 CD8^+^ T cells and their wild-type counterparts sorted on day 21 p.i. Principal-component analysis (PCA) demonstrated a distinct transcriptomic profile of sgCtrl compared with *Arid2*- and *Pbrm1*-deleted CD8^+^ T cells (Figures 2I and 2J). Furthermore, gene set enrichment analysis (GSEA) indicated that pathways involved in cell cycle progression, cell proliferation, DNA repair, and chromatin stability were significantly upregulated, whereas pathways related to apoptosis were downregulated in PBAF-deleted CD8^+^ T cells (Figures 2K–2M). One of the gene sets consistently upregulated following *Arid2* and *Pbrm1* deletion was the targets of E2F TFs, including *Cenpf, Cdc25b, Top2a*, and Cdkn1a (Figures S3F and S3G), which are actively involved in cell cycle regulation, DNA synthesis, DNA repair, and apoptosis.^52^ Taken together, our transcriptomic and cellular analyses suggest that PBAF restricts the expansion of virus-specific CD8^+^ T cells by regulating cell proliferation and apoptosis pathways at the late phase of chronic infection.

### PBAF promotes T_PRO_-to-T_EXH_ transition during chronic LCMV infection

**Figure 3.**
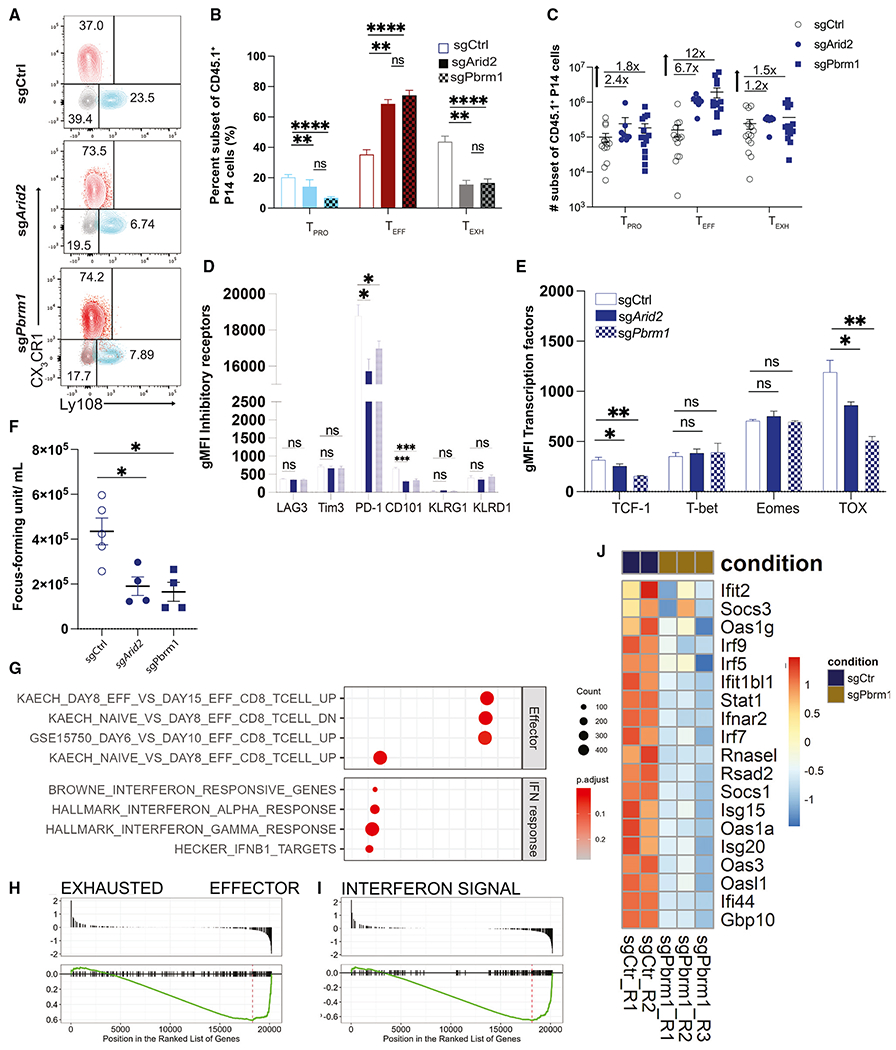
PBAF promotes the transition of T_PRO_ to T_EXH_ by regulating IFN-I response (A) Representative flow plots and summary data showing the frequency of the three subsets of control and *Arid2*- or *Pbrm1*-deleted CD45.1^+^CD8^+^ T cells 21 days p.i. (B and C) Representative and summary plot displaying the frequency and numbers of the three subsets within control or *Arid2* and *Pbrm1* guide RNA (gRNA) transduced P14 CD8^+^ T cells. (D and E) Summary data showing gMFI of LAG3, Tim3, PD-1, 2B4, KLRD1, KLRG1, TIGIT, TCF1, Tbet, Eomes, and TOX in CD45.1^+^ CD8 T cells. (F) Plot displaying viral load in the sera from experimental mice reciving control or *Arid2*- or *Pbrm1*-deleted T cells. (G) Bulk RNA-seq GSEA results showing pathways significantly up- or downregulated in Pbrm1-deficient P14 T cells during LCMV Cl13 infection. (H and I) Enrichment plots showing representative gene sets identified in the GSEA of Pbrm1-deficient P14 T cells. (J) Heatmap of IFN pathway-related genes with differential expression between control and Pbrm1-deficient conditions. Summary data (mean ± SEM) are pooled from at least 2 independent experiments with at least 4 mice/group per experiment. (F) is from one independent experiment with at least 4 mice/group. Data are representative of three independent experiments. (G–J) Four replicates were included in each condition. *p < 0.05, **p < 0.01, ***p < 0.0001.

To elucidate whether abrogation of the PBAF complex using CRISPR/ribonucleoprotein (RNP) recapitulates the phenotypic changes observed in Arid2-deficient CD8^+^ T cells in the bone marrow chimeric model (Figure 1), we deleted *Arid2* and *Pbrm1* in P14 CD8^+^ T cells and assessed their cellular responses on day 21 p.i. In accordance with our mixed BMC experiments, we observed a significant increase (~2-fold) in the frequency of T_EFF_ cells and a proportional reduction of T_PRO_ and T_EXH_ subsets (Figures 3A and 3B). Furthermore, consistent with the increased expansion of virus-specific CD8^+^ T cells, loss of PBAF led to an increase in the overall absolute numbers of cells in all three subsets, with the highest magnitude increase of T_EFF_ compared with T_PRO_ and T_EXH_ subsets (Figure 3C). These results further support our finding regarding the role of PBAF in restricting expansion and T_PRO_-to-T_EFF_ differentiation during chronic viral infection. To ensure that the change in phenotype is restricted to the loss of PBAF in adoptively transferred P14 cells, we assessed the endogenous CD8^+^ T cells specific to the LCMV GP_276-286_ epitope measured by the H-2D^b^ tetramer. On day 21 p.i., no difference in the frequency and subset distribution of the GP_276_-specific CD8^+^ T cells was observed in the recipient mice that received *Arid2*- and *Pbrm1*-deleted P14 cells compared with their control counterparts (Figure S3E).

Interestingly, loss of PBAF in CD8^+^ T cells did not alter the expression of phenotypic markers related to T cell exhaustion, such as TIM-3 and LAG3, and effector function, such as KLRG1 and KLRD1 (Figure 3D). The expression of PD-1 was modestly lower in *Arid2*- and *Pbrm1*-deleted CD8^+^ T cells (Figure 3D). Consistently, we observed a significant reduction in the expression of another exhaustion marker, CD101, in *Arid2*- and *Pbrm1*-deleted CD8^+^ T cells, suggesting reduced terminal exhaustion in the absence of PBAF. Although we observed a similar capacity of degranulation, IFNγ, and granzyme B production between the wild-type and *Arid2*- or *Pbrm1*-deleted P14 CD8^+^ T cells (Figures S3G and S3H), the *Arid2*- and *Pbrm1*-deleted P14 CD8^+^ T cell-recipient mice had a significantly lower serum viral load compared with their wild-type counterparts (Figure 3F). This suggests that a sheer increase of virus-responding T_EFF_ CD8^+^ T cells in the absence of PBAF most likely accounts for the improved viral control. Finally, loss of Arid2 and Pbrm1 resulted in a significant reduction in expression of the TFs TCF-1 and TOX, whereas the expression of T-bet and Eomes was largely unchanged (Figure 3E).

To dissect the possible mechanisms by which the PBAF complex regulates CD8^+^ T cell differentiation, we performed bulk RNA-seq on *Pbrm1*- and *Arid2*-deleted P14 CD8^+^ T cells and their wild-type counterparts sorted on day 21 p.i. GSEA showed that *Pbrm1*-deficient CD8^+^ T cells exhibited higher effector CD8^+^ T cell signatures at the whole transcriptional level (Figures 3G and 3H). Notably, one of the key pathways significantly downregulated in *Pbrm1*- and *Arid2*-deleted CD8^+^ T cells was the type I IFN response pathway. Genes involved in IFN response, including *Ifi44, Gbp10, Usp18, Oas3, Stat1*, and *Irf7*, were most downregulated in *Pbrm1*- or *Arid2*-deleted CD8^+^ T cells (Figures 3J and S3F). Collectively, our data suggest that PBAF promotes transcription of key exhaustion associated programs such as type I IFN (IFN-I) to promote T_PRO_-to-T_EXH_ differentiation of the virus-specific CD8^+^ T cells.

### PBAF regulates chromatin accessibility associated with cell survival and IFN response

**Figure 4.**
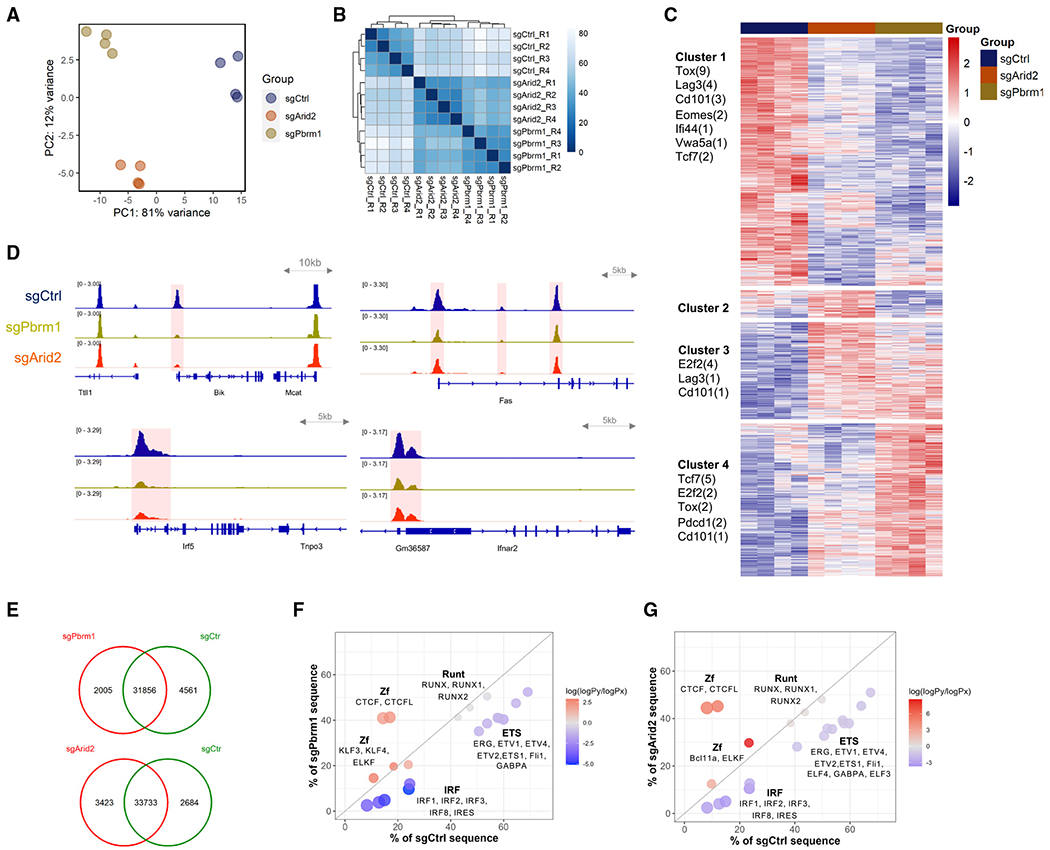
ATAC-seq analysis reveals chromatin accessibility regulated by PBAF (A) PCA plot showing the top two principles delineating the genome-wide chromatin accessibility variations among sgPbrm1, sgArid2, and sgCtrl P14 T cells at day 21 post-LCMV Cl13 infection. Four replicates were included in each condition. (B) Correlation heatmap showing the similarity of chromatin accessibility profiles across ATAC-seq samples. (C) Heatmap showing unsupervised clustering of differentially accessible chromatin regions. Number of peaks associated with genes of interest are listed under each cluster. (D) IGV tracks showing the open chromatin regions associated with representative genes involved in apoptosis (*Bik* and *Fas*) and IFN response (*Irf5* and *Ifnar2*). Differentially accessible regions (p-adjust < 0.05) are highlighted in red. (E) Venn plots showing the number of overlapped and condition-specific chromatin-accessible regions identified by occupancy analysis. (F and G) Motifs most significantly enriched in sgPbrm1-specific (F) or sgArid2-specific (G) versus sgCtrl-specific ATAC-seq peak sets identified in the occupancy analysis. Dot position shows the percentage of condition-specific sequences that contain the motifs. Dot color represents the normalized enrichment score.

PBAF as a chromatin remodeling complex is known to regulate gene expression by orchestrating chromatin-accessible regions (ChARs) and gene transcription activity.^18,40,46,53–56^ To illustrate the genome-wide changes of ChARs regulated by the PBAF complex, we performed assay for transposase-accessible chromatin sequencing (ATAC-seq) on sgCtrl and *Pbrm1*- and *Arid2*-deleted P14 CD8^+^ T cells fluorescence-activated cell sorted (FACS) from LCMV Cl13-infected recipient mice at day 21 p.i. PCA and correlation heatmap showed large similarities between the chromatin landscapes of *Pbrm1*- and *Arid2*-deficient CD8^+^ T cells, which were distinct from the sgCtrl group (Figures 4A and 4B). Unsupervised clustering of all significantly different peaks (adjusted p value [p-adjust] < 0.05) showed a cluster of ChARs that lost accessibility when *Pbrm1* or *Arid2* was deleted in CD8^+^ T cells (Figure 4C, cluster 1). Consistent with the flow cytometry and RNA-seq analysis, ChARs associated with the T_EXH_ signature such as *Tox* and *Cd101* were less accessible in Pbrm1- and Arid2-deficient groups compared with their control counterparts (Figure 4C, cluster 1). More strikingly, ChARs associated with multiple apoptosis-regulating genes including Bik and Fas showed significant reduction in accessibility (Figure 4D, top panels). In line with the reduced IFN-I response pathways observed by RNA-seq, reduced chromatin accessibility was also observed at the genetic loci associated with IFN response genes, including Irf5 and Ifnar2 (Figure 4D, bottom panels).

To delineate TFs whose binding activities could be regulated by PBAF, we compared ChARs from *Pbrm1*- and *Arid2*-deleted CD8^+^ T cells with those from control counterparts. Compared with the control, 2,005 and 4,561 ChARs were gained and lost, respectively, in the absence of *Pbrm1*, and 3,423 and 2,684 ChARs were gained and lost, respectively, in the absence of *Arid2* (Figure 4E). With these differentially accessible regions, we performed motif enrichment analysis. Strikingly, CTCF/CTCFL motifs were highly enriched in the peak sets that gained accessibility when *Pbrm1* or *Arid2* was CRIPSR deleted (Figures 4F and 4G). The CTCF motif was found in over 40% of the regions that were only open in Pbrm1- or Arid2-deficient CD8^+^ T cells. Furthermore, IRF motifs (IRF1, IRF2, IRF3, IRF4, IRF8, ISRE) were much more enriched in the lost ChARs, which suggests that PBAF may maintain the accessibility of IRFs. These observations suggest a potential PBAF- and IFN-involved mechanism that may contribute to CD8^+^ T cell exhaustion. Overall, the analysis of chromatin accessibility suggests that PBAF regulates chromatin permissiveness at gene loci, regulating the survival and exhaustion of CD8^+^ T cells during chronic viral infection.

### Single-cell multiomics reveals PBAF-regulated exhaustion and proliferation programs in a subset-specific manner

**Figure 5.**
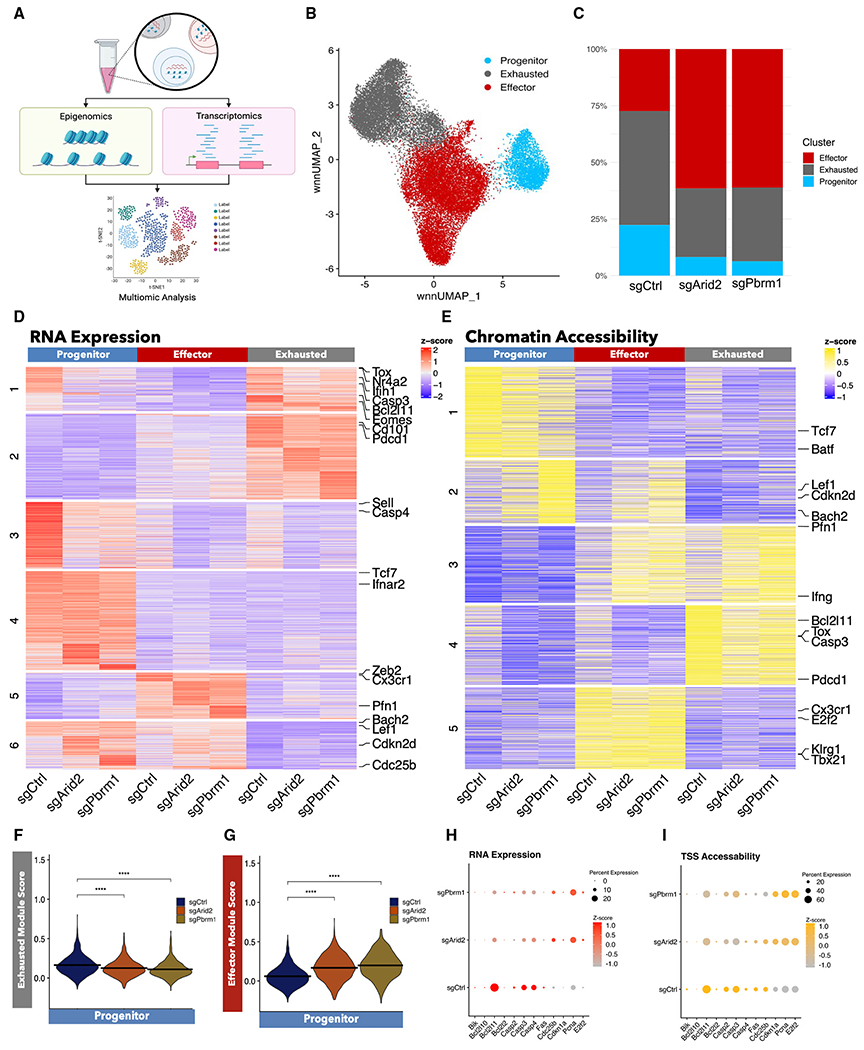
Single-cell multiomics reveals PBAF-regulated exhaustion and proliferation programs in a subset-specific manner (A) Experimental design for multimodal analysis of the same cell with both single-nucleus RNA (snRNA) and scATAC-seq following chronic infection with LCMV Cl13. (B) Weighted nearest-neighbor uniform manifold approximation and projection (wnnUMAP) plot of splenic GP33^+^ CD8^+^ T cells from three mice from 21 days post-LCMV Cl13 infection. Each dot represents one cell, and cells are colored by cluster identity. (C) Bar graph showing cluster distribution frequencies of each sample. (D) Heatmap of differentially expressed genes for sgCtrl, sgArid2, and sgPbrm1 conditions for each cluster with K-means clustering. (E) Heatmap of differentially expressed chromatin regions for sgCtrl, sgArid2, and sgPbrm1 conditions for each cluster with K-means clustering. (F) Module scores of the top 100 differentially expressed genes from previously identified exhausted splenic CD8^+^ T cells for sgCtrl, sgArid2, and sgPbrm1 in the progenitor cluster. (G) Module scores of the top 100 differentially expressed genes from previously identified effector splenic CD8^+^ T cells for sgCtrl, sgArid2, and sgPbrm1 in the progenitor cluster. (F and G) p values determined by two-sided Wilcoxon rank-sum test. *p < 0.05, **p < 0.01, ***p < 0.0001. (H and I) Dot plot showing expression of proliferation markers. Dot size denotes the number of cells with a particular gene expressed, and intensity of dot color indicates the expression level of RNA expression (H) and transcription start site (TSS) accessibility (I).

To gain further insights into how the PBAF complex regulates dynamic chromatin structure changes and examine how PBAF deficiency alters the transcriptional and epigenetic profiles of CD8^+^ T cells, we performed single-cell multiomics analysis on PBAF-deficient CD8^+^ T cells and their wild-type counterparts (Figure 5A). Consistent with previously published work^15^ our multiome data revealed three distinct clusters: progenitor (*Tcf7, Il7r*, and *Slamf6*), effector (*Zeb2*), and exhausted (*Lag3*, *Havcr2, Nr4a2*), which was consistent for both gene expression (Figure S4B) and chromatin accessibility (Figure S4C). Consistent with our previous findings, we found an increased proportion of the effector cluster, with a corresponding decrease in the proportion of both the progenitor and exhausted clusters, in the absence of Arid2 or Pbrm1 (Figure 5C).

To examine patterns of gene regulation across each subset with and without PBAF, we found all differentially expressed genes and differentially accessible chromatin regions with an p-adjust of less than 0.05. We then applied K-means clustering to sort these genes and chromatin regions into distinct patterns of expression on a heatmap (Figures 5D and 5E). A cluster of genes (RNA heatmap cluster 1) and peaks (accessibility heatmap cluster 4) that are classically associated with the T_EXH_ subset such as *Tox* and *Nr4a2* and the IFN-I pathway was differentially enriched between the sgCtrl and PBAF-deleted CD8^+^ T cells (Figures 5D and 5E). Interestingly, the T_EXH_ signature was expressed in the progenitor cluster, but only in wild type. To quantify this expression pattern more accurately, we took the top 100 differentially expressed genes within the progenitor, effector, and exhausted subsets from our previously published dataset (GEO: GSE129139) and applied them to *Arid2*- and *Pbrm1*-deleted CD8^+^ T cells using Seurat’s module score function (Figure S4D). Notably, PBAF-deficient CD8^+^ T cells had a lower exhausted module score in the T_PRO_ cluster than the wild-type control cells (Figure 5F). Conversely, in the absence of PBAF, the progenitor cells had an increased effector module score (Figure 5G). Overall, this indicates that PBAF plays an important role in maintaining the exhaustion signature starting from the progenitor stage.

Lastly, we examined how PBAF alters apoptotic and proliferative profiles using the same K-means clustering approach of differentially expressed genes and chromatin regions (Figures 5D and 5E). Notably, we observed a decrease in the expression of apoptotic markers (*Bcl2l11*, *Casp3*) within the PBAF-deficient progenitor cluster (Figures 5D and 5E), whereas other apoptotic markers (*Bik*, *Casp4*) had decreased expression regardless of the cluster (Figures 5H, 5I, and S4E). Furthermore, there was a cluster of genes (RNA expression cluster 6) and peaks (peak accessibility heatmap cluster 2) associated with proliferation-related markers (*Cdc25a*, *Cdkn2d*) that was enriched in PBAF-deficient cells within the progenitor and effector subsets (Figures 5D and 5E). Moreover, this group of genes also had progenitor-associated TFs, such as *Lef1* and *Bach2*, which possibly suggests that in the absence of PBAF, effector cells may retain some progenitor-like properties (Figures 5D and 5E). Collectively, our results suggest that during chronic viral infection, PBAF maintains the permissive chromatin landscape of T_EXH_ genes to determine the T_EXH_ cell fate from T_PRO_. Furthermore, PBAF determine the chromatin accessibility of genes associated with apoptosis and proliferation in the T_PRO_ subset to regulate the clonal expansion of CD8^+^ T cells at the late phase of chronic infection.

### PBAF-dependent TF motif accessibility regulates CD8^+^ T cell differentiation and proliferation

**Figure 6.**
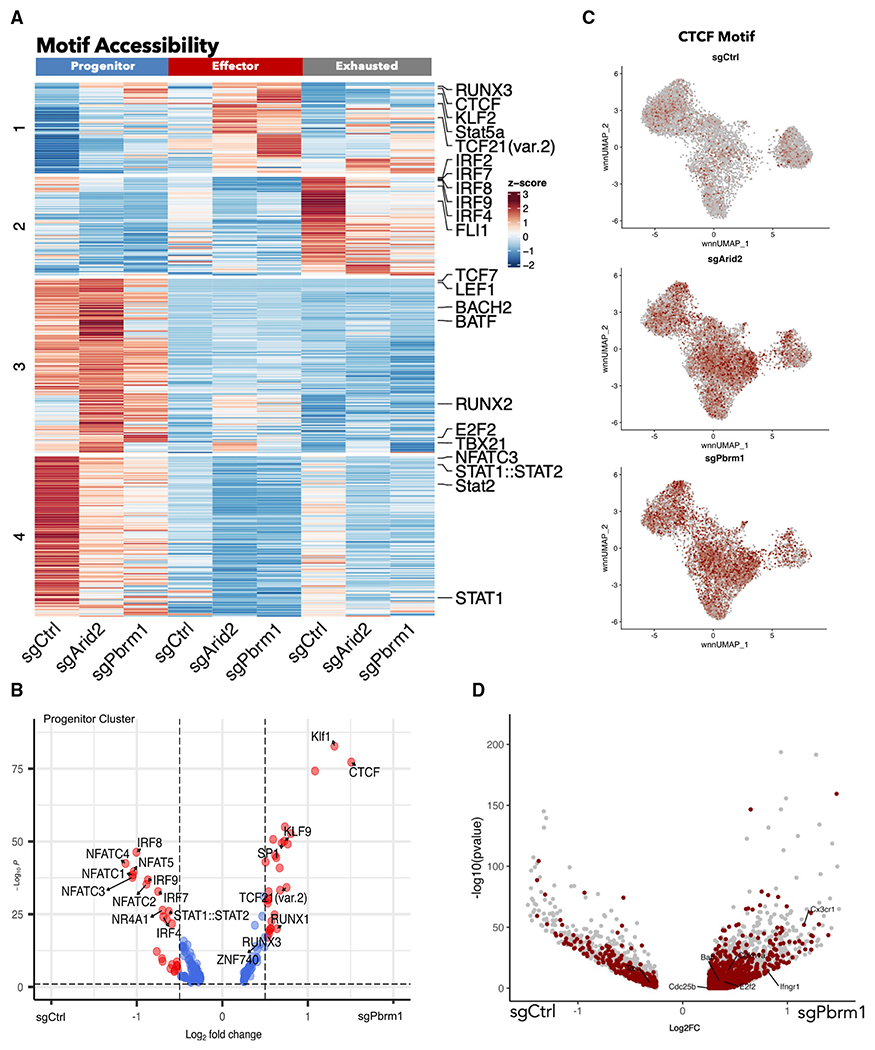
PBAF modulates motif accessibility (A) Heatmap of differentially accessible TF motifs for sgCtrl, sgArid2, and sgPbrm1 conditions for each cluster with K-means clustering. (B) Volcano plot showing motifs that are differentially accessible between sgPbrm1 and sgCtrl in the progenitor cluster. (C) wnnUMAP of CTCF motif accessibility represented by chromVar deviation scores. (D) Volcano plot showing differentially accessible peaks that contain the CTCF motif between sgArid2 and sgCtrl.

To further delineate the possible PBAF-modulated transcriptional programs that regulate the cell-fate decision of virus-specific CD8^+^ T cells, TF motif accessibility was examined with chromVAR deviation scores. These scores identify TF binding motifs enriched in differentially accessible chromatin regions, which we use for examination of how TF activity may change across clusters and conditions.^57^ First, we confirmed that chromVAR’s definition of motif accessibility aligned well with previously known subset-specific enhancer programs. Specifically, TCF-1, T-bet, and IRF4 binding motifs were enriched in the progenitor, effector, and exhausted clusters, respectively (Figure S5A). In order to understand the role of PBAF in driving T cell exhaustion, we next assessed how motif accessibility is modulated by the PBAF complex. To do this, we found significantly different chomVAR scores and clustered them using K-means clustering (Figure 6A). We observed the most striking differences within the progenitor cluster among wild-type and *Arid2*- and *Pbrm1*-deleted groups. Of note, we found a group of motifs associated with exhaustion, such as IRF2,^58^ IRF4,^25^ IRF7,^26^ and FLI1^59^ (motif accessibility heatmap cluster 2) (Figure 6A) present in the exhausted cluster. The group of exhausted motifs showed a reduced accessibility in the PBAF-deficient T_EXH_ subset. Interestingly, this group of exhaustion motifs was also present in the progenitor subset, but only in the sgCtrl group. Additionally, a cluster containing STAT1 and STAT2 (motif accessibility heatmap cluster 4), which are both known TFs involved in IFN-I response,^58,60,61^ showed enriched accessibility in the progenitor subset (Figure 6A). Interestingly, this cluster of motifs lost accessibility in the sg*Arid2* or sg*Pbrm1* groups, suggesting that PBAF modulates the IFN-I response, starting from the progenitor stage. Given the largest differences taking place within the progenitor population, we next examined the motif accessibility within the progenitor cluster specifically and identified major differences between wild-type and PBAF-deficient CD8^+^ T cells (Figures 6A, 6B, and S5B). Consistent with previously reports showing that NFAT is a critical TF for establishing an exhaustion cell fate,^28^ a reduction in NFAT motif accessibility (NFATC1, NFATC2, NFATC3, NFATC4, NFAT5) was observed in the absence of PBAF (Figures 6A, 6B, and S5B). Conversely, we found a group of motifs that had increased accessibility in the absence of PBAF, mostly within the effector clusters (chromatin accessibility heatmap cluster 1) (Figure 6A). Specifically, we observed increased accessibility of Stat5a and Runx3 motifs (Figures 6A, 6B, and S5B). Importantly, Stat5 signaling can epigenetically rewire exhausted CD8^+^ T cells to a more durable effector-like state under chronic antigenic stress.^62^ Additionally, Runx3 has been shown to drive effector differentiation.^59^ Overall, these data suggest that PBAF regulates the accessibility of TF families in a subset-dependent manner, which is necessary for promoting T cell exhaustion while restricting effector differentiation.

Interestingly, we found that CTCF had the largest increase in motif accessibility across the *Arid2*- and *Pbrm1*-deleted T cells when compared with wild-type CD8^+^ T cells across all clusters (Figure 6C). In CD8^+^ T cells, CTCF has been shown to regulate the genomic reorganization necessary for CD8^+^ effector function through genes, such as *Tbx21, Ifng*, and *Klrg1*.^63^ To investigate this idea further, we examined the differentially accessible regions that had a CTCF-binding motif present. We found that in the absence of PBAF, several effector-related genes such as *Ifngr1, Batf*, and *Cx3cr1* all had more accessible chromatin regions that contained a CTCF motif (Figure 6D). Additionally, it has also recently been described that TCF-1 and its homolog LEF1 bind to CTCF to undergo homeostatic proliferation in response to antigen.^64^ Interestingly, we noticed that some cells in the effector cluster continued to express *Tcf7* and *Lef1*, specifically in the absence of PBAF (Figures S5E and S5F). Prior studies on terminally differentiated T cells indicated that ectopic expression of TCF-1 could induce a more progenitor like state.^65^ Lastly, in the PBAF-deficient CD8^+^ T cells, CTCF-binding motifs were found in more accessible chromatin regions with cell cycle-related genes such as *E2f2, Cdc20, Cdc25b*, and *Cdkn1a* (Figures 6D, S5C, and S5D), suggesting the possible mechanisms of increased cellular proliferation associated with reorganized chromatin structure. Together, these observations indicate that a fraction of PBAF-deficient effector cells retain “progenitor” properties and obtain some capacity to restrain from terminal differentiation through 3D-chromatin structure changes.

### Loss of PBAF promotes expansion and limits exhaustion of tumor-specific CD8^+^ T cells to confer tumor control

**Figure 7.**
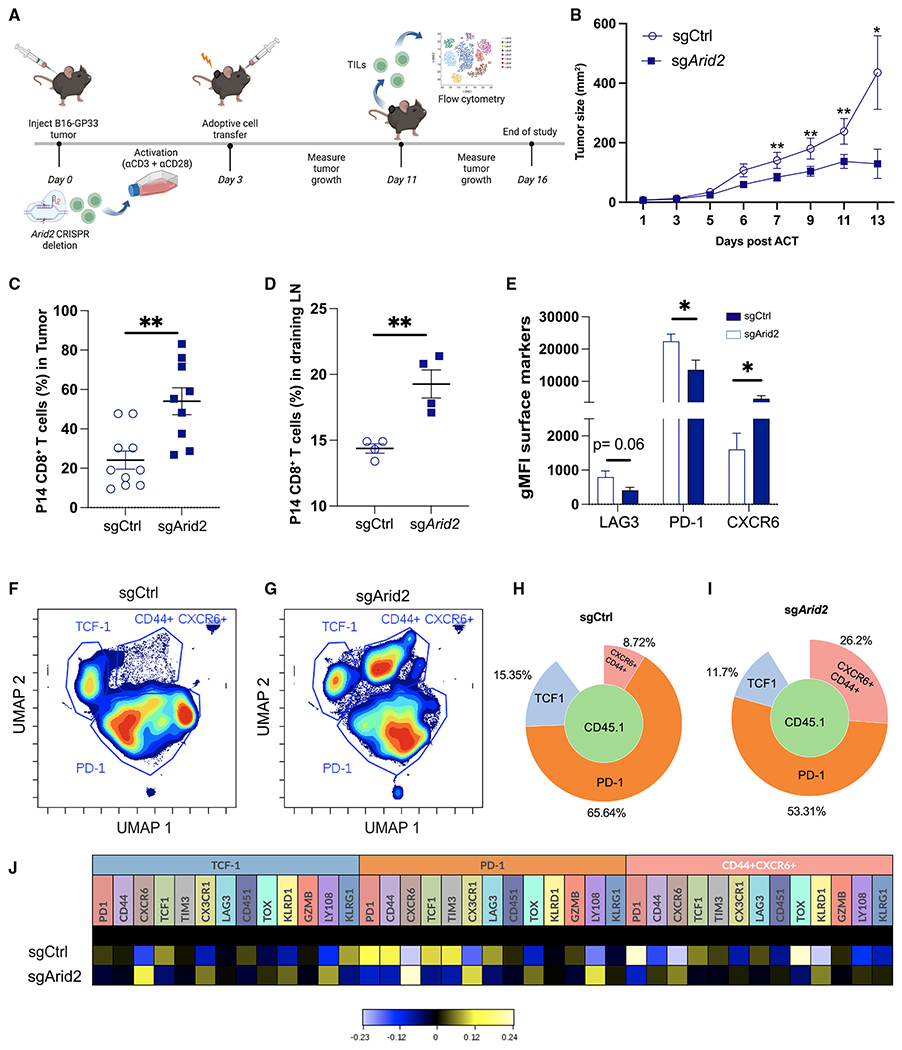
Loss of PBAF promotes expansion and limits T cell exhaustion to control tumor (A) Experimental design. (B) Tumor growth curves; of note, summary data are from (n = 9) sgCtrl transduced cell recipients and (n = 13) sg*Arid2* transduced cell recipients. sgCtrl transduced cell recipient mice that did not developed >10% of tumor volume (n = 1) were excluded from the analysis. (C and D) Summary plot showing the frequency of CD45.1^+^ CD8^+^ T cells in the tumor and draining lymph node at day 8 post-ACT. (E) FlowJo analysis showing summary data showing the per-cell expression (gMFI) of LAG3, PD1, and CXCR6 in CD45.1^+^ CD8^+^ T cells. (F-J) The phenotype of CD45.1^+^ CD8^+^ T cells in the tumor was assessed by high-dimensional spectral flow cytometry on 12 markers using Cytobank (STAR Methods). (F and G) Unbiased UMAP displaying clusters identified four clusters following concatenation of CD45.1^+^ CD8 T cells: (n = 6) sgCtrl transduced cells and (n = 6) sg*Arid2* transduced cells. (H and I) Frequency of the three populations displayed using Population Sunburst. (J) Heatmap showing the mean expression of each marker in gated population. Data (mean ± SEM) in (C) and (E) are pooled and are from at least 3 mice/group/experiment and are representative of at least 3 independent experiments. (D) is from one independent experiment with 4 mice/group. Heatmaps were generated using hyperbolic arcsine (arcsinh) transformation against the mean expression of the combined concatenated samples. *p < 0.05, **p < 0.01, ***p < 0.0001.

Harnessing the effects of perturbation of PBAF on T cell expansion and exhaustion, we decided to test if deleting PBAF in CD8^+^ T cells could improve tumor control. To do this, we employed the B16-F10 melanoma tumor cells expressing the LCMV GP_33-41_ epitope (referred to as B16-GP_33_ hereafter) (Figure 7A). Remarkably, by day 7 after adoptive cell transfer (ACT), the recipients of *Arid2*-deleted P14 CD8^+^ T cells had a significantly lower tumor burden compared with the control group (Figure 7B). To investigate the tumor-specific CD8^+^ T cell response, we isolated CD8^+^ T cells on day 8 after ACT and observed a significant increase in the frequency and cell number of *Arid2*-deficient CD8^+^ P14 cells in the tumor and draining lymph nodes (Figures 7B–7D, S6A, and S6B). Strikingly, *Arid2*-deficient P14 cells displayed a significant reduction in the expression of PD-1 and LAG3 and a significant increase in the expression of CXCR6 (Figure 7E). Together, these findings indicate that the loss of Arid2 generates less exhausted, highly proliferative tumor-infiltrating effector CD8^+^ T cells, resulting in improved tumor control.

To determine whether the loss of PBAF complex activity promotes effector differentiation and limits exhaustion as observed in chronic viral infection, we performed multidimensional flow cytometry to fully characterize the heterogeneity of tumor-specific CD8^+^ T cells. We observed an overall reduction in expression of TCF-1, PD-1, and TIM-3 and increased expression of effector genes such as CXCR6 and CX_3_CR1 in *Arid2*-deleted CD8^+^ T cells (Figure S6C). Notably, tumor-specific CD8^+^ T cells grouped distinctly into three major clusters as visualized by uniform manifold approximation and projection (UMAP) (Figures 7F and 7G). Our analysis identified the presence of a stem-like cluster that exhibited high expression of TCF-1 (Figure S6D), an exhaustion subset expressing PD-1 (Figure S6E), and an effector-like subset that coexpressed CXCR6 and CD44 (Figures S6F and S6G). We observed a significant increase in the frequency of the effector like CXCR6^+^CD44^+^ cluster in the tumor from *Arid2*-deleted CD8^+^ T cell recipients compared with the control (26.2% versus 8.72%) (Figures 7H and 7I). The increased effector differentiation was accompanied by a reduction in the frequency of PD-1^+^ and TCF-1^+^ clusters in *Arid2*-deleted CD8^+^ T cells (Figures 7H and 7I). Intriguingly, Arid2-deleted CD8^+^ T cells coexpressed CX_3_CR1, CXCR6, and KLRD1 in the TCF-1^+^ progenitor cluster, suggesting that loss of PBAF results in retention of “progenitor” properties in effector cells (Figure 7J). Moreover, the PD-1^+^ cluster in *Arid2*-deficient CD8^+^ T cells displayed reduced expression of TIM-3, LAG3, and PD-1, further suggesting that perturbation of PBAF sufficiently limits T cell exhaustion (Figure 7J). Lastly, the loss of Arid2 promoted the expression of KLRD1 and inhibited TCF1 and TOX expression in the CXCR6^+^CD44^+^ cluster, further indicating an increased effector profile of tumor-specific CD8^+^ T cells following PBAF deletion (Figure 7J).

Finally, to compare the wild-type and *Arid2*-deleted CD8^+^ T cell response independent of the tumor volume, we cotransferred an equal number of congenic sgCtrl and sgArid2 P14 CD8^+^ T cells into B16-GP_33_-bearing recipient mice (Figure S6H). At day 8 after ACT, we isolated tumor-infilitrating lymphocytes (TILs) and assessed CD8^+^ T cell response. Notably, Arid2-deleted CD8^+^ T cells exhibited a significant increase in the number and frequency of tumor-specific Arid2-deficient CD8^+^ T cells compared with their wild-type counterparts (Figures S6I–S6K). Furthermore, Arid2-deficient CD8^+^ T cells consisted lower frequency of PD-1^+^TOX^+^ CD8^+^ T cells compared with the sgCtrl group. This suggests that given the same tumor microenvironment, Arid2 deficiency confers higher proliferative capacity and reduced terminal exhaustionin tumor-responding CD8^+^ T cells than their wild-type counterparts. Of note, consistent with the chronic infection model, we did not notice any significant difference in the expression of granzyme B between the control group and the Arid2-deleted CD8^+^T cells Figure S6N). Collectively, our data demonstrate that the PBAF complex plays an important role in regulating expansion and promoting exhaustion in tumor-specific CD8^+^T cells. Perturbation of PBAF complex activity in CD8^+^ T cells renders higher proliferative capacity and reduced exhaustion, which makes PBAF an attractive target for cancer immunotherapy.

## DISCUSSION

In this study, we identified a previously unappreciated role of the PBAF complex in CD8^+^ T cell differentiation during chronic viral infection and cancer. We demonstrated that PBAF as a chromatin remodeling complex restrained clonal expansion and promoted T cell exhaustion at the expense of effector cell differentiation during chronic LCMV infection. Our multiomics analyses demonstrated that the PBAF-mediated epigenetic landscape regulated cell cycle progression and apoptosis and favored exhaustion-related transcription programs, such as the IFN-I response. To harness this new knowledge, we performed ACT experiments with *Arid2*-deleted CD8^+^ T cells and found increased tumor control mediated by heightened effector cell proliferation and limited exhaustion. Overall, these findings improve our understanding of how PBAF-regulated chromatin changes affect the cell-fate decision during CD8^+^ T cell differentiation. Targeting PBAF complex activity could lead to therapeutic designs to overcome T cell exhaustion in treating chronic infection and cancer.

Despite playing a critical role in T cell development, proliferation, and activation, the role of BAF complexes in CD8^+^ T cell differentiation has been understudied. Recently loss of the cBAF complex was reported to preserve the memory potential of CD8^+^ T cells and prevent terminal differentiation.^47,48^ This distinct function of the cBAF complex from the PBAF complex (this study) suggests a lineage-specific role of the BAF complexes in regulating the chromatin organization to determine CD8^+^ T cell fate. Additionally, the ncBAF complex was recently identified as the key regulator of Foxp3 and T_reg_ lineage stability.^46^ It remains to be investigated whether the ncBAF complex also plays distinct role in regulating the differentiation trajectories of CD8^+^ T cells during chronic infection and cancer. Given the availability of pharmacological BAF inhibitors and their reported CD8^+^-intrinsic and -extrinsic tumor suppression, elucidating molecular mechanisms of how BAF complexes coordinately regulate CD8^+^ T cell differentiation will likely gain more attraction in the future.

Our single-cell multiomics analysis revealed that CTCF gene expression, chromatin accessibility, and its motif binding score were noticeably shifted from T_PRO_ and T_EXH_ subsets to the T_EFF_ subset in the absence of PBAF. Coincident with this redistribution of the CTCF-binding motif was increased chromatin accessibility and gene expression of *Tcf7* and *Lef1* in the T_EFF_ subset when PBAF activity is abrogated. Given that *Tcf7* and *Lef1* should normally be silenced while T_PRO_ transitions to the terminal TEFF subset, this unexpected gene activity suggests that the reorganized chromatin structures may favor effector cell differentiation without losing some progenitor features, such as CTCF-TCF-1-dependent proliferative potential as previously reported.^64^ This may also account for the increased clonal size observed in PBAF-deficient CD8^+^ T cells. Interestingly, although constitutive CTCF-binding sites are well regarded for maintaining invariable chromatin architecture, more recent studies indicate that a considerable fraction of CTCF occupancy is dynamic (i.e., variable across cell types) and lineage specific.^66–69^ The dynamic CTCF binding was reported to colocalize with lineage-specific TFs at the key *cis*-regulatory elements (CREs), governing hematopoiesis^69^ and CD8^+^ T cell homeostasis.^64^ More intriguingly, the recently identified association of BRG1 with CTCF and cohesion complex^70,71^ suggests that SWI/SNF chromatin remodeling complexes could regulate the accessibility of dynamic CTCF-binding sites.^38,72^ Thus, it would be exciting to investigate if PBAF could regulate CD8^+^ T cell differentiation through the CTCF-regulated structure changes of CREs.

Our high-dimensional flow cytometry analysis identified three clusters of tumor-infiltrating CD8^+^ T cells that phenotypically resembled the three major subsets observed during chronic viral infection. It is, however, important to note that there were differences in CD8^+^ T cell subset definition between cancer and chronic viral infection. Recently, CD8^+^ T cells expressing CXCR6 have been identified as highly proliferative and functional tumor-infiltrating CD8^+^ T cells that promote tumor control. Using this knowledge, we identified an effector-like subset expressing CXCR6, along with high KLRD1 and CD44 expression.^73,74^ Despite the different surface receptor, we observed similar phenotypic changes in PBAF-deleted CD8^+^ T cells from an LCMV Cl13 infection and tumor model.

### Limitations of the study

Our research indicates that there are slight variations in the characteristics of *Arid2*- and *Pbrm1*-deleted CD8^+^ T cells. We could not accurately determine deletion efficiency between *Arid2* and *Pbrm1* gRNAs due to the lack of commercially available western blot antibodies to detect Arid2. Additionally, we were unable to obtain data to demonstrate the genomic localization of the PBAF complex, which would enable us to identify the precise molecular mechanism by which the PBAF complex regulated expansion and T_PRO_-to-T_EXH_ differentiation. Therefore, the changes in the chromatin accessibility observed following the loss of PBAF cannot be used as conclusive evidence of PBAF-mediated epigenetic changes. Another caveat of this study is that the heterogeneity of CD8^+^ T cells is limited to only three defined subsets, and recent advances have demonstrated the presence of an intermediate subset^26^ and a new progenitor subset expressing CD62L.^20^ Therefore, it is likely that the PBAF complex plays a bigger role in determining the cell fate of CD8^+^ T cells during chronic infection.

## STAR★METHODS

### RESOURCE AVAILABILITY

#### Lead contact

Further information and requests for resources and reagents should be directed to and will be fulfilled by the Lead Contact, Weiguo Cui (Weiguo.cui@northwestern.edu).

#### Materials availability

No new reagents were generated in this study.

#### Data and code availability

- The Single Cell Multiome ATAC + Gene Expression, Bulk RNA sequencing and Bulk ATAC sequencing data have been deposited at Gene Expression Omnibus (GEO): GSE and are publicly available as of the date of publication. Accession numbers are listed in the key resources table.
- This paper does not report original code.
- Any additional information required to reanalyze the data reported in this paper is available from the lead contact upon request.

### EXPERIMENTAL MODEL AND STUDY PARTICIPANT DETAILS

#### Mice, mixed bone marrow chimeras and LCMV infection

Four-to eight-week-old female C57BL/6 and female C57BL/6 CD45.1 congenic mice were purchased from Charles River. Female Vav-cre^+^; *Arid2*^*flox/flox*^ mice were kindly provided by Dr. Nan Zhu (Medical college of Wisconsin, Milwaukee, WI). Mixed bone marrow chimeras were generated by reconstituting lethally irradiated CD45.1/CD45.2 CD8α^−/−^ mice (10 male and 5 female) with bone marrow from CD45.1 CD8^−/−^ and wildtype CD45.2 or Arid2^−/−^ Cd45.2 donors mixed at 7:3 ratio (Figures S2A–S2C). All donor mice were female. To establish chronic infection, 2 × 10^6^ PFU LCMV Cl13 was intravenously injected into each mouse. LCMV Cl13 was prepared by a single passage on BHK21 cells and viral titers were determined by plaque formation assay on Vero cells. All animal husbandry and experiments were approved by the Institutional Animal Care and Use Committee (IACUC) at the Medical College of Wisconsin and Northwestern University.

### METHOD DETAILS

#### CRISPR/Cas9 RNP transfection and adoptive cell transfer

CRIPSR/RNP transfection was performed as previously described.^51^ RNP electroporation was performed with naive CD8^+^ T cells (for LCMV Cl13 infection), or total CD8^+^ T cells (for tumor experiments) isolated from spleens of donor P14 mice using EasySep immunomagnetic negative selection kits from STEMCELL. Briefly, Cas9 (Alt-R S.p. Cas9 Nuclease, IDT) and sgRNAs (Synthego) were combined and incubated at RT for 10 min. For each target, two sgRNAs was used to increase knockout efficiency. Electroporation was performed using the 4D-NucleofectorTM 4 Core Unit and P3 primary cell 4D-NucleofectorTM5 X kit S with program DN100. Following the electroporation, cells were kept in an incubator for 10 min at 37°C. For LCMV Cl13 infection studies, 2,500 cells were immediately adoptively transferred to naive C57BL/6 recipient mice via *i.v.* injection followed by LCMV Cl13 infection. For tumor studies, the cells were activated with anti-CD3 and anti-CD28 for 3 days and 1x10^6^ cells were adoptively transferred to separate groups of tumor-bearing mice. For co-transfer tumor experiments, cells were mixed at a 1:1 ratio (1x10^6^) before adoptive transfer.

#### Tumor cell lines and tumor inoculation

B16-F10 cells from ATCC were used to generate B16-GP_33_ tumor cell line expressing the LCMV Cl13 GP_33-41_ peptide. Tumor cells were cultured in DMEM media (Corning) supplemented with 10% (v/v) FBS and Geneticin selective antibiotics at a final concentration of 200 μg/mL. Melanoma tumors were established by subcutaneously injecting 0.5 × 10^5^ B16-GP_33_ cells at the right flank of C57BL/6 mice. For tumor growth experiments, mice were randomly assigned to different treatment groups. Tumor growth was monitored by calipers every other day, and tumor volume was calculated as [length × (width)^2^]/2.

#### Immune cell isolation from tumors

Tumors were dissected, cut into 2–3 mm sections, and digested in complete RPMI media containing 10% FBS, 0.7 mg/mL collagenase I (Worthington, Lakewood, NJ, USA), 100 μg/mL bovine pancreatic DNase type I, grade II (Sigma-Aldrich, St. Louis, MO, USA), and 5 mM MgCl2 (Sigma-Aldrich) for 1 h at 37°C while shaking. Tumors were then passed through a cell strainer and immune cells were isolated via gradient centrifugation with LymphoPrep (Stem Cell Technologies, Vancouver, BC, Canada, USA).

#### Flow cytometry

Lymphocytes isolated from spleen, blood, and lymph nodes were stained with H-2^D^b/GP_33_ (MHC class I tetramer) or congenic markers (CD45.1/CD45.2), together with antibodies against cell surface antigens for 30–60 min at 4°C. For Granzyme B and cytokine staining, cells were then fixed with the paraformaldehyde Fixation Buffer (Biolegend) for 20 min at RT. For transcription factor staining, cells were fixed with True-Nuclear Transcription Factor Buffer Set (Biolegend) overnight at 4°C. Intracellular and transcription factor stains were performed in Intracellular Staining Permeabilization Wash Buffer (Biolegend). Flow cytometry data were acquired on an LSRII, FACSCelesta or Cytek Aurora flow cytometer and analyzed using FlowJo or Cytobank.

#### Focus forming assay for virus titer quantification

Serum was isolated, snap-frozen on dry ice, and subsequently stored in an −80°C freezer. Vero cells were seeded in a 96-well plate at a density of 30,000 cells/well and cultured overnight. Serum samples were added to Vero cells (1:25 dilution) and incubated at 37°C, 5% CO2 for ~20 h. Infected cells were detected by probing with rat anti-LCMV nucleoprotein InVivo MAb clone VL-4 (BioXCell BED106), followed by goat anti-rat IgG2a-FITC (BethylA110-109F). Clusters of infected cells (foci) were counted by IncuCyte S3 Live-Cell Analysis System and reported as focus forming units (FFU).

#### TIDE assay

The TIDE assay was performed as previously described. Briefly, DNA was extracted from cells (DNeasy Blood and Tissue kit, QIAgen catalog no. 69506) and PCR was used to amplify the expected sgRNA target site, which was then purified (QIAquick PCR Purification kit, QIAgen catalog no. 28106) and analyzed by Sanger sequencing.

#### Bulk RNA sequencing and data analysis

CD45.1^+^ CD8 P14 T cell were FACS-sorted from the spleens of LCMV Cl13-infected mice at day 21 p.i. Total RNA was purified with RNeasy Plus Micro Kit (Qiagen) and used for preparing RNA-seq libraries following the SMART-seq2 protocol^82^ and sequenced on an Illumina NextSeq 500 sequencer. Raw sequencing data was first processed by *nf-core/rnaseq* pipeline (v3.8.1)^83^ with the default settings. Sequencing reads were aligned to GRCm38 mouse genome by *Salmon.*^84^ Differential analysis was then performed with *DEseq2* (v1.36.0) (Love MI, 2014). Gene Set Enrichment Analysis (GSEA) was performed with *clusterProfiler* (v4.4.4)^85^ and gene set database *msigdbr* (v7.5.1).^86,87^ *ggplot2* (v3.4.0) was used for plotting.

#### ATAC sequencing and data analysis

A total of 50,000 FACS-sorted P14 T cells from LCMV Cl13-infected mice at day 21 p.i were used for ATAC-seq library construction following the Omni-ATAC protocol.^88^ Paired-end sequencing of the libraries was performed on an Illumina NextSeq 500 sequencer. Raw sequencing data were first processed by *nf-core/atacseq* pipeline (v1.2.2)^83^ with the default settings. Sequencing reads were aligned to GRCm38 mouse genome by *BWA*.^89^ *MACS2*^90^ was used for peak calling with a threshold of FDR >0.05 and consensus peaks that were found in at least two replicates were kept for downstream analysis. Differential analysis was then performed with *DEseq2* (v1.36.0).^91^ Preprocessed data were analyzed with *DiffBind* (v3.6.5)^92^ to identify the open chromatin regions uniquely accessible in the consensus peaks sets of each condition. The identified condition specific peak sets were then exported in bed file format for motif analysis and gene annotation using *Homer* (v4.1.0).^93^ Peak tracks were visualized by *IGV viewer*.^94,95^

#### Single cell multiomic sequencing

CD45.1^+^ CD8 P14 cells were FACS-sorted from the spleen and inguinal lymph nodes of mice at day 21 post LCMV Cl13 infection. Nuclei were isolated and processed with the Chromium Single Cell Multiome ATAC + Gene Expression Reagent Kit following manufacture’s manual (10x genomics). Two separate sets of libraries, Gene expression (GEX) and ATAC, were generated from each sample. GEX libraries were sequenced on an Illumina NextSeq 500 sequencer and ATAC libraries were sequenced on an Illumina NovaSeq 6000 sequencer by Linda T. and John A. Mellowes Center for Genomic Sciences and Precision Medicine at Medical College of Wisconsin.

#### Single cell multiomic sequencing analysis

Raw sequencing data were downloaded from Illumina BaseSpace. The “mkfastq” and ’count” functions from *CellRanger-arc* (v2.0.2) (10x Genomics) were used to demultiplex and covert data to a gene-barcode matrix. Reads were aligned to the mm10 reference genome. Analysis was performed in R (v4.2.1) using the package *Seurat* (v4.2.0)^77,78,79^ and *Signac*.^96^ The package *ggplot2* (v3.3.6) was used to generate figures while the package *tidyverse* (v1.3.2) used to organize data. Quality control was performed on each modality independently, using standard approaches for RNA and ATAC-seq data. Quality control filtrated out low quality cells with a high percentage of mitochondrial genes in the transcriptome (>6%). High quality cells were determined to have between 200 and 2000 unique genes and between 3000 and 70000 peaks, which removed background noise and doublets. Log normalization and variable-feature identification were performed for each sample individually. We then used the *FindIntegrationAnchors* function to overlay the datasets. When scaling gene expression values, cell cycle scores were regressed. T cell receptor genes were removed from variable features. Chromatin accessibility counts were normalized using term frequency inverse document frequency (TF-IDF). Dimensionality of chromatin data was reduced by singular value decomposition. The resultant LSI (latent semantic indexing) and RNA anchors were used to integrate across samples with IntegrateEmbeddings() function. We then calculated Weighted Nearest Neighbors (WNN) and visualized this through dimensionality reduction by uniform manifold approximation (UMAP). To analyze motifs, position frequency matrices were downloaded from the JASPAR2020 database.^97^ Motif activities per cell were calculated using the *RunChromVAR* function in Signac.

### QUANTIFICATION AND STATISTICAL ANALYSIS

Statistical analysis was performed using Prism 9 for macOS. A two-tailed Students’ t-test was used to calculate statistical significance between two independent conditions. A p-value of <0.05 was considered significant.

## Supplementary Material

1

## Figures and Tables

**Table:** KEY RESOURCES TABLE

REAGENT or RESOURCE	SOURCE	IDENTIFIER
Antibodies		
PE LCMV I-A(b) GP66-77 tetramer	NIH Tetramer Core Facility	https://tetramer.yerkes.emory.edu
PE or APC LCMV D^b^GP33 tetramer	NIH Tetramer Core Facility	https://tetramer.yerkes.emory.edu
PE LCMV D^b^GP276 tetramer	NIH Tetramer Core Facility	https://tetramer.yerkes.emory.edu
PE LCMV D^b^NP396 tetramer	NIH Tetramer Core Facility	https://tetramer.yerkes.emory.edu
BV711 anti-mouse CD4	BioLegend	Cat# 100549, RRID:AB_11219396
BV510 anti-mouse/human CD44	BioLegend	Cat# 103044, RRID:AB_2650923
APC anti-mouse CD185 (CXCR5)	BioLegend	Cat# 145506, RRID: AB_2561970
PE/Cyanine7 anti-mouse CX3CR1	BioLegend	Cat#149016, RRID: AB_2565700
APC/Cyanine7 anti-mouse CD279 (PD-1)	BioLegend	Cat#, 135224 RRID: AB_2563523
PE/Dazzle 594 anti-mouse CD186 (CXCR6)	BioLegend	Cat# 151117, RRID:AB_2721700
PE/Dazzle 594 anti-mouse/human CD45R/B220 Antibody	BioLegend	Cat# 103258, RRID:AB_2564053
APC anti-mouse CD138 (Syndecan-1)	Biolegend	Cat# 142505, RRID:AB_10960141
FITC anti-MU/HU GL7 Antigen (T/B Cell Act. Marker)	BioLegend	Cat# 144604, RRID:AB_2561697
APC/Cyanine7 anti-mouse IgM	Biolegend	Cat# 406515, RRID:AB_10690815
PE anti-mouse CD95 (Fas)	BioLegend	Cat# 152608, RRID:AB_2632902
Brilliant Violet 421 anti-mouse IgD	Biolegend	Cat# 405725, RRID:AB_2562743
Brilliant Violet 711 anti-mouse CD8a	Biolegend	Cat# 100748, RRID:AB_2562100
APC anti-mouse CD223 (LAG-3)	Biolegend	Cat# 125209, RRID:AB_10639935
PE/Cyanine7 anti-mouse CD186 (CXCR6)	Biolegend	Cat# 151119, RRID:AB_2721670
CD366 (TIM3) Monoclonal Antibody (RMT3-23), FITC,	eBioscience	Cat# 11-5870-82, RRID:AB_2688129
TOX Antibody, anti-human/mouse, PE, REAfinity^™^	Miltenyi Biotec	Cat# 130-120-716, RRID:AB_2801780
APC anti-T-bet	Biolegend	Cat# 644814, RRID:AB_10901173
PE anti-T-bet	Biolegend	Cat# 644810, RRID:AB_2200542
EOMES Monoclonal Antibody (Dan11mag), PE-Cyanine7	eBioscience	Cat# 25-4875-82, RRID:AB_2573454
FITC Donkey anti-rabbit IgG (min. x-reactivity)	Biolegend	Cat# 406403, RRID:AB_893531
PE/Cyanine7 anti-mouse IFN-gamma	Biolegend	Cat# 505826, RRID:AB_2295770
APC anti-mouse/rat TNF-alpha	Biolegend	Cat# 506108, RRID:AB_2721315
APC/Cyanine7 anti-mouse CD107a (LAMP-1)	Biolegend	Cat# 121616, RRID:AB_10643268
PE/Dazzle 594 anti-human/mouse Granzyme B Recombinant	Biolegend	Cat# 372215, RRID:AB_2728382
APC/Cyanine7 anti-mouse/human CD44	BioLegend	Cat# 103028, RRID:AB_830785
PE/Cyanine7 anti-mouse CD279 (PD-1)	BioLegend	Cat# 135216, RRID:AB_10689635
PE/Dazzle 594 anti-mouse CX3CR1	BioLegend	Cat# 149014, RRID:AB_2565698
Pacific Blue anti-mouse Ly108	BioLegend	Cat# 134608, RRID:AB_2188093
Pacific Blue anti-mouse CD45.2	BioLegend	Cat# 109820, RRID:AB_492872
APC/Cyanine7 anti-mouse/human KLRG1 (MAFA)	Biolegend	Cat# 138425, RRID:AB_2566553
BV480 Rat Anti-Mouse CD8a	BDbiosciences	Cat# 566096, RRID:AB_2739500
BV510 anti-mouse CD366 (TIM-3)	BDbiosciences	Cat# 747625, RRID:AB_2744191
Brilliant Violet 605(TM) anti-mouse CX3CR1	Biolegend	Cat# 149027, RRID:AB_2565937
BV650 anti-mouse TIGIT	BDbiosciences	Cat# 744213, RRID:AB_2742062
Brilliant Violet 711(TM) anti-mouse CD186 (CXCR6)	Biolegend	Cat# 151111, RRID:AB_2721558
Brilliant Violet 785(TM) anti-mouse CD45.1	Biolegend	Cat# 110743, RRID:AB_2563379
FITC anti-rat CD90/mouse CD90.1 (Thy-1.1)	Biolegend	Cat# 202504, RRID:AB_1595653
PE/Cyanine5 anti-mouse/human CD44	Biolegend	Cat# 103009, RRID:AB_312960
BUV395 anti-mouse CD44	BDbiosciences	Cat# 740215, RRID:AB_2739963
PE/Cyanine7 anti-mouse CD94	Biolegend	Cat# 105509, RRID:AB_2632663
Bacterial and virus strains		
LCMV Clone 13	Rafi Ahmed, PhD	Grown in house
Chemicals, peptides, and recombinant proteins		
Brefeldin A Solution (1,000X)	Biolegend	Cat#420601
Fixation Buffer	Biolegend	Cat#420801
KAVYNFATM (GP_3341_) peptide	GenScript	RP20-257
True Nuclear Transcription Factor Buffer Set	Biolegend	Cat#424401
Live/Dead Fixable Aqua Kit	ThermoFisher	Cat# L34957
Collagenase, Type 1	Worthington	Cat# LS004194
DNase I	Milipore Sigma	Cat# 10104159001
Critical commercial assays		
EasySep Mouse CD8+ T cell isolation Kit	Stem Cell	Cat#19853
EasySep^™^ Mouse Naive CD8+ T cell Isolation Kit	Stem Cell	Cat#19858
Chromium Next GEM Single Cell Multiome ATAC + Gene Expression Reagent Bundle, 4 rxns	10X Genomics	Cat# 1000285
Dynabeads^™^ MyOne^™^ SILANE	10x Genomics	Cat# PN-2000048
Library Construction Kit	10x Genomics	Cat# PN-1000190
Dual Index Kit TT Set A	10x Genomics	Cat# PN-1000215
SPRIselect Reagent Kit	Beckman Coulter	Cat#B23318
Kappa NGS quantification kit	KAPABiosystems	Cat#KK4824
NextSeq 500/550 High Output Kit v2.5 (150 cycles)	Illumina	Cat#20024907
HSD5000 ScreenTape	Agilent	Cat# 5067-5592
Agencourt AMPure XP	Beckman Coulter	Cat# A63880
Deposited data		
Single cell ATAC + RNAseq, Bulk ATCAseq and Bulk RNAseq from CD45.1^+^ P14 CD8^+^ T cells, day 21 post-LCMV Cl13 infection	This paper	GSE222346
scRNAseq from GP33+ CD8 T cells, day 30 post LCMV Cl13 infection	Zander et al.^15^	GSE129139
Experimental models: Organisms/strains		
C57BL/6 mice	Charles River	N/A
CD45.1 congenic mice	Charles River	N/A
Vav-cre^+^; *Arid2*^*flox/flox*^ mice	Previous study	Bluemn et al.^75^
B16-D^B^GP33 (B16_gp33_)	Prévost-Blondel et al.^76^	Grown in house
Oligonucleotides		
GCCGTTTAAGAAGATCCCTG (Arid2_gRNA#1)	This paper	Synthego
TCCGCCTAAAGTAGTGACTC (Arid2_gRNA#2)	This paper	Synthego
TGATCCATACTGAAGTGCCA (Pbrm1_gRNA#1)	This paper	Synthego
TCCAGAAAACTTTCGCGATG (Pbrm1_gRNA#2)	This paper	Synthego
Software and algorithms		
Cell Ranger 6.0	10x Genomics	https://support.10xgenomics.com/single-cell-gene-expression/software/pipelines/latest/installation
Seurat 4.0.6	Butler et al. and Stuart et al.^77,78^	https://satijalab.org/seurat/
FlowJo 10.7.1	Tree Star	N/A
Prism 9	Graphpad Software	N/A
Cytobank	Beckman Coulter	https://support.cytobank.org/hc/en-us
Signac 1.9.0	Stuart et al.^79^	https://stuartlab.org/signac/
Tidyverse 1.3.2	Wickham et al.^80^	https://www.tidyverse.org/
Complex Heatmap 3.17	Gu et al.^81^	https://github.com/jokergoo/ComplexHeatmap
Biorender	N/A	Biorender.com

## Highlights

- PBAF complex restricts expansion of virus-specific CD8^+^ T cells during chronic infection
- Loss of PBAF promotes T_PRO_-to-T_EFF_ transition during chronic infection and cancer
- PBAF regulates motif accessibility of transcription factors that drive T cell exhaustion
- PBAF-deficient CD8^+^ T cells confer higher viral and tumor control

## References

[1] Wherry EJ, and Kurachi M (2015). Molecular and cellular insights into T cell exhaustion. Nat. Rev. Immunol 15, 486–499. 10.1038/nri3862. 26205583 PMC4889009

[2] Virgin HW, Wherry EJ, and Ahmed R (2009). Redefining chronic viral infection. Cell 138, 30–50. 10.1016/j.cell.2009.06.036. 19596234

[3] McLane LM, Abdel-Hakeem MS, and Wherry EJ (2019). CD8 T cell exhaustion during chronic viral infection and cancer. Annu. Rev. Immunol 37, 457–495. 10.1146/annurev-immunol-041015-055318. 30676822

[4] Zajac AJ, Vance RE, Held W, Sourdive DJ, Altman JD, Raulet DH, and Ahmed R (1999). Impaired anti-viral T cell responses due to expression of the Ly49A inhibitory receptor. J. Immunol 163, 5526–5534. 10553080

[5] Wherry EJ, Blattman JN, Murali-Krishna K, van der Most R, and Ahmed R (2003). Viral persistence alters CD8 T-cell immunodominance and tissue distribution and results in distinct stages of functional impairment. J. Virol 77, 4911–4927. 10.1128/jvi.77.8.4911-4927.2003. 12663797 PMC152117

[6] Beltra JC, Manne S, Abdel-Hakeem MS, Kurachi M, Giles JR, Chen Z, Casella V, Ngiow SF, Khan O, Huang YJ, (2020). Developmental relationships of four exhausted CD8(+) T cell subsets reveals underlying transcriptional and epigenetic landscape control mechanisms. Immunity 52, 825–841.e8. 10.1016/j.immuni.2020.04.014. 32396847 PMC8360766

[7] Chen Z, Ji Z, Ngiow SF, Manne S, Cai Z, Huang AC, Johnson J, Staupe RP, Bengsch B, Xu C, (2019). TCF-1-Centered transcriptional network drives an effect or versus exhausted CD8 T cell-fate decision. Immunity 51, 840–855.e5. 10.1016/j.immuni.2019.09.013. 31606264 PMC6943829

[8] He R, Hou S, Liu C, Zhang A, Bai Q, Han M, Yang Y, Wei G, Shen T, Yang X, (2016). Follicular CXCR5- expressing CD8(+) T cells curtail chronic viral infection. Nature 537, 412–428. 10.1038/nature19317. 27501245

[9] Hudson WH, Gensheimer J, Hashimoto M, Wieland A, Valanparambil RM, Li P, Lin JX, Konieczny BT, Im SJ, Freeman GJ, (2019). Proliferating transitory T cells with an effector-like transcriptional signature emerge from PD-1(+) stem-like CD8(+) T cells during chronic infection. Immunity 51, 1043–1058.e4. 10.1016/j.immuni.2019.11.002. 31810882 PMC6920571

[10] Im SJ, Hashimoto M, Gerner MY, Lee J, Kissick HT, Burger MC, Shan Q, Hale JS, Lee J, Nasti TH, (2016). Defining CD8+ T cells that provide the proliferative burst after PD-1 therapy. Nature 537, 417–421. 10.1038/nature19330. 27501248 PMC5297183

[11] Kanev K, Wu M, Drews A, Roelli P, Wurmser C, von Hösslin M, and Zehn D (2019). Proliferation-competent Tcf1 + CD8 T cells in dysfunctional populations are CD4 T cell help independent. Proc. Natl. Acad. Sci. USA 116, 20070–20076. 10.1073/pnas.1902701116. 31530725 PMC6778176

[12] Leong YA, Chen Y, Ong HS, Wu D, Man K, Deleage C, Minnich M, Meckiff BJ, Wei Y, Hou Z, (2016). CXCR5(+) follicular cytotoxic T cells control viral infection in B cell follicles. Nat. Immunol 17, 1187–1196. 10.1038/ni.3543. 27487330

[13] Miller BC, Sen DR, Al Abosy R, Bi K, Virkud YV, LaFleur MW, Yates KB, Lako A, Felt K, Naik GS, (2019). Subsets of exhausted CD8(+) T cells differentially mediate tumor control and respond to checkpoint blockade. Nat. Immunol 20, 326–336. 10.1038/s41590-019-0312-6. 30778252 PMC6673650

[14] Utzschneider DT, Charmoy M, Chennupati V, Pousse L, Ferreira DP, Calderon-Copete S, Danilo M, Alfei F, Hofmann M, Wieland D, (2016). T cell factor 1-expressing memory-like CD8(+) T cells sustain the immune response to chronic viral infections. Immunity 45, 415–427. 10.1016/j.immuni.2016.07.021. 27533016

[15] Zander R, Schauder D, Xin G, Nguyen C, Wu X, Zajac A, and Cui W (2019). CD4(+) T cell help is required for the formation of a cytolytic CD8(+) T cell subset that protects against chronic infection and cancer. Immunity 51, 1028–1042.e4. 10.1016/j.immuni.2019.10.009. 31810883 PMC6929322

[16] Raju S, Xia Y, Daniel B, Yost KE, Bradshaw E, Tonc E, Verbaro DJ, Kometani K, Yokoyama WM, Kurosaki T, (2021). Identification of a T-bet(hi) quiescent exhausted CD8 T cell subpopulation that can differentiate into TIM3(+)CX3CR1(+) effectors and memory-like cells. J. Immunol 206, 2924–2936. 10.4049/jimmunol.2001348. 34088768 PMC8642473

[17] Henning AN, Roychoudhuri R, and Restifo NP (2018). Epigenetic control of CD8(+) T cell differentiation. Nat. Rev. Immunol 18, 340–356. 10.1038/nri.2017.146. 29379213 PMC6327307

[18] Belk JA, Daniel B, and Satpathy AT (2022). Epigenetic regulation of T cell exhaustion. Nat. Immunol 23, 848–860. 10.1038/s41590-022-01224-z. 35624210 PMC10439681

[19] Yao C, Lou G, Sun HW, Zhu Z, Sun Y, Chen Z, Chauss D, Moseman EA, Cheng J, D’Antonio MA, (2021). BACH2 enforces the transcriptional and epigenetic programs of stem-like CD8(+) T cells. Nat. Immunol 22, 370–380. 10.1038/s41590-021-00868-7. 33574619 PMC7906956

[20] Tsui C, Kretschmer L, Rapelius S, Gabriel SS, Chisanga D, Knöpper K, Utzschneider DT, Nüssing S, Liao Y, Mason T, (2022). MYB orchestrates T cell exhaustion and response to checkpoint inhibition. Nature 609, 354–360. 10.1038/s41586-022-05105-1. 35978192 PMC9452299

[21] Gautam S, Fioravanti J, Zhu W, Le Gall JB, Brohawn P, Lacey NE, Hu J, Hocker JD, Hawk NV, Kapoor V, (2019). The transcription factor c-Myb regulates CD8(+) T cell stemness and antitumor immunity. Nat. Immunol 20, 337–349. 10.1038/s41590-018-0311-z. 30778251 PMC6489499

[22] Chen J, López-Moyado IF, Seo H, Lio CWJ, Hempleman LJ, Sekiya T, Yoshimura A, Scott-Browne JP, and Rao A (2019). NR4A transcription factors limit CAR T cell function in solid tumours. Nature 567, 530–534. 10.1038/s41586-019-0985-x. 30814732 PMC6546093

[23] Liu X, Wang Y, Lu H, Li J, Yan X, Xiao M, Hao J, Alekseev A, Khong H, Chen T, (2019). Genome-wide analysis identifies NR4A1 as a key mediator of T cell dysfunction. Nature 567, 525–529. 10.1038/s41586-019-0979-8. 30814730 PMC6507425

[24] Paley MA, Kroy DC, Odorizzi PM, Johnnidis JB, Dolfi DV, Barnett BE, Bikoff EK, Robertson EJ, Lauer GM, Reiner SL, and Wherry EJ(2012). Progenitor and terminal subsets of CD8+ T cells cooperate to contain chronic viral infection. Science 338, 1220–1225. 10.1126/science.1229620. 23197535 PMC3653769

[25] Man K, Gabriel SS, Liao Y, Gloury R, Preston S, Henstridge DC, Pellegrini M, Zehn D, Berberich-Siebelt F, Febbraio MA, (2017). Transcription factor IRF4 promotes CD8(+) T cell exhaustion and limits the development of memory-like T cells during chronic infection. Immunity 47, 1129–1141.e5. 10.1016/j.immuni.2017.11.021. 29246443

[26] Kasmani MY, Zander R, Chung HK, Chen Y, Khatun A, Damo M, Topchyan P, Johnson KE, Levashova D, Burns R, (2023). Clonal lineage tracing reveals mechanisms skewing CD8+ T cell fate decisions in chronic infection. J. Exp. Med 220, e20220679. 10.1084/jem.20220679. 36315049 PMC9623343

[27] Anderson DA, Ou F, Kim S, Murphy TL, and Murphy KM (2022). Transition from cMyc to L-Myc during dendritic cell development coordinated by rising levels of IRF8. J. Exp. Med 219, e20211483. 10.1084/jem.20211483. 34958351 PMC8713298

[28] Martinez GJ, Pereira RM, Äijö T, Kim EY, Marangoni F, Pipkin ME, Togher S, Heissmeyer V, Zhang YC, Crotty S, (2015). The transcription factor NFAT promotes exhaustion of activated CD8(+) T cells. Immunity 42, 265–278. 10.1016/j.immuni.2015.01.006. 25680272 PMC4346317

[29] Shin H, Blackburn SD, Intlekofer AM, Kao C, Angelosanto JM, Reiner SL, and Wherry EJ (2009). A role for the transcriptional repressor blimp-1 in CD8(+) T cell exhaustion during chronic viral infection. Immunity 31, 309–320. 10.1016/j.immuni.2009.06.019. 19664943 PMC2747257

[30] Alfei F, Kanev K, Hofmann M, Wu M, Ghoneim HE, Roelli P, Utzschneider DT, von Hoesslin M, Cullen JG, Fan Y, (2019). TOX reinforces the phenotype and longevity of exhausted T cells in chronic viral infection. Nature 571, 265–269. 10.1038/s41586-019-1326-9. 31207605

[31] Khan O, Giles JR, McDonald S, Manne S, Ngiow SF, Patel KP, Werner MT, Huang AC, Alexander KA, Wu JE, (2019). TOX transcriptionally and epigenetically programs CD8(+) T cell exhaustion. Nature 571, 211–218. 10.1038/s41586-019-1325-x. 31207603 PMC6713202

[32] Scott AC, Dündar F, Zumbo P, Chandran SS, Klebanoff CA, Shakiba M, Trivedi P, Menocal L, Appleby H, Camara S, (2019). TOX is a critical regulator of tumour-specific T cell differentiation. Nature 571, 270–274. 10.1038/s41586-019-1324-y. 31207604 PMC7698992

[33] Yao C, Sun HW, Lacey NE, Ji Y, Moseman EA, Shih HY, Heuston EF, Kirby M, Anderson S, Cheng J, (2019). Single-cell RNA-seq reveals TOX as a key regulator of CD8(+) T cell persistence in chronic infection. Nat. Immunol 20, 890–901. 10.1038/s41590-019-0403-4. 31209400 PMC6588409

[34] Chen Y, Zander RA, Wu X, Schauder DM, Kasmani MY, Shen J, Zheng S, Burns R, Taparowsky EJ, and Cui W (2021). BATF regulates progenitor to cytolytic effector CD8(+) T cell transition during chronic viral infection. Nat. Immunol 22, 996–1007. 10.1038/s41590-021-00965-7. 34282329 PMC9258987

[35] Giles JR, Ngiow SF, Manne S, Baxter AE, Khan O, Wang P, Staupe R, Abdel-Hakeem MS, Huang H, Mathew D, (2022). Shared and distinct biological circuits in effector, memory and exhausted CD8(+) T cells revealed by temporal single-cell transcriptomics and epigenetics. Nat. Immunol 23,1600–1613. 10.1038/s41590-022-01338-4. 36271148 PMC10408358

[36] Daniel B, Yost KE, Hsiung S, Sandor K, Xia Y, Qi Y, Hiam-Galvez KJ, Black M, J Raposo C, Shi Q, (2022). Divergent clonal differentiation trajectories of T cell exhaustion. Nat. Immunol 23, 1614–1627. 10.1038/s41590-022-01337-5. 36289450 PMC11225711

[37] Alfert A, Moreno N, and Kerl K (2019). The BAF complex in development and disease. Epigenet. Chromatin 12, 19. 10.1186/s13072-019-0264-y. PMC6427853 30898143

[38] Centore RC, Sandoval GJ, Soares LMM, Kadoch C, and Chan HM (2020). Mammalian SWI/SNF chromatin remodeling complexes: emerging mechanisms and therapeutic Strategies. Trends Genet. 36, 936–950. 10.1016/j.tig.2020.07.011. 32873422

[39] Kadoch C, and Crabtree GR (2015). Mammalian SWI/SNF chromatin remodeling complexes and cancer: mechanistic insights gained from human genomics. Sci. Adv 1, e1500447. 10.1126/sciadv.1500447. 26601204 PMC4640607

[40] Mittal P, and Roberts CWM (2020). The SWI/SNF complex in cancer - biology, biomarkers and therapy. Nat. Rev. Clin. Oncol 17, 435–448. 10.1038/s41571-020-0357-3. 32303701 PMC8723792

[41] Chi TH, Wan M, Zhao K, Taniuchi I, Chen L, Littman DR, and Crabtree GR (2002). Reciprocal regulation of CD4/CD8 expression by SWI/SNF-like BAF complexes. Nature 418, 195–199. 10.1038/nature00876. 12110891

[42] Jeong SM, Lee C, Lee SK, Kim J, and Seong RH (2010). The SWI/SNF chromatin-remodeling complex modulates peripheral T cell activation and proliferation by controlling AP-1 expression. J. Biol. Chem 285, 2340–2350. 10.1074/jbc.M109.026997. 19910461 PMC2807292

[43] Osipovich O, Cobb RM, Oestreich KJ, Pierce S, Ferrier P, and Oltz EM (2007). Essential function for SWI-SNF chromatin-remodeling complexes in the promoter-directed assembly of Tcrb genes. Nat. Immunol 8, 809–816. 10.1038/ni1481. 17589511

[44] Lee S, Kim J, Min H, and Seong RH (2020). RORgammat-driven T(H) 17 cell differentiation requires epigenetic control by the Swi/Snf chromatin remodeling complex. iScience 23, 101106. 10.1016/jJsci.2020.101106. 32434140 PMC7235640

[45] Letimier FA, Passini N, Gasparian S, Bianchi E, and Rogge L (2007). Chromatin remodeling by the SWI/SNF-like BAF complex and STAT4 activation synergistically induce IL-12Rbeta2 expression during human Th1 cell differentiation. EMBO J. 26, 1292–1302. 10.1038/sj.emboj.7601586. 17304212 PMC1817634

[46] Loo CS, Gatchalian J, Liang Y, Leblanc M, Xie M, Ho J, Venkatraghavan B, Hargreaves DC, and Zheng Y (2020). A genome-wide CRISPR screen reveals a role for the non-canonical nucleosome-remodeling BAF complex in Foxp3 expression and regulatory T cell function. Immunity 53, 143–157.e8. 10.1016/jJmmuni.2020.06.011. 32640256 PMC7341821

[47] Belk JA, Yao W, Ly N, Freitas KA, Chen YT, Shi Q, Valencia AM, Shifrut E, Kale N, Yost KE, (2022). Genome-wide CRISPR screens of T cell exhaustion identify chromatin remodeling factors that limit T cell persistence. Cancer Cell 40, 768–786.e7. 10.1016/j.ccell.2022.06.001. 35750052 PMC9949532

[48] Guo A, Huang H, Zhu Z, Chen MJ, Shi H, Yuan S, Sharma P, Connelly JP, Liedmann S, Dhungana Y, (2022). cBAF complex components and MYC cooperate early in CD8(+) T cell fate. Nature 607, 135–141. 10.1038/s41586-022-04849-0. 35732731 PMC9623036

[49] Yan Z, Cui K, Murray DM, Ling C, Xue Y, Gerstein A, Parsons R, Zhao K, and Wang W (2005). PBAF chromatin-remodeling complex requires a novel specificity subunit, BAF200, to regulate expression of selective interferon-responsive genes. Genes Dev. 19, 1662–1667. 10.1101/gad.1323805. 15985610 PMC1176002

[50] Georgiades P, Ogilvy S, Duval H, Licence DR, Charnock-Jones DS, Smith SK, and Print CG (2002). VavCre transgenic mice: a tool for mutagenesis in hematopoietic and endothelial lineages. Genesis 34, 251–256. 10.1002/gene.10161. 12434335

[51] Nüssing S, House IG, Kearney CJ, Chen AXY, Vervoort SJ, Beavis PA, Oliaro J, Johnstone RW, Trapani JA, and Parish IA (2020). Efficient CRISPR/Cas9 gene editing in uncultured naive mouse T cells for in vivo studies. J. Immunol 204, 2308–2315. 10.4049/jim-munol.1901396. 32152070

[52] Dimova DK, and Dyson NJ (2005).The E2F transcriptional network: old acquaintances with new faces. Oncogene 24, 2810–2826. 10.1038/sj.onc.1208612. 15838517

[53] Hodges C, Kirkland JG, and Crabtree GR (2016). The many roles of BAF (mSWI/SNF) and PBAF complexes in cancer. Cold Spring Harb. Perspect. Med 6, a026930. 10.1101/cshperspect.a026930. 27413115 PMC4968166

[54] Roberts CWM, and Orkin SH (2004). The SWI/SNF complex–chromatin and cancer. Nat. Rev. Cancer 4, 133–142. 10.1038/nrc1273. 14964309

[55] Tolstorukov MY, Sansam CG, Lu P, Koellhoffer EC, Helming KC, Alver BH, Tillman EJ, Evans JA, Wilson BG, Park PJ, and Roberts CWM (2013). Swi/Snf chromatin remodeling/tumor suppressor complex establishes nucleosome occupancy at target promoters. Proc. Natl. Acad. Sci. USA 110, 10165–10170. 10.1073/pnas.1302209110. 23723349 PMC3690861

[56] Bayona-Feliu A, and Aguilera A (2021). The SWI/SNF complex, transcription-replication conflicts and cancer: a connection with high therapeutic potential. Mol. Cell. Oncol 8, 1976582. 10.1080/23723556.2021.1976582. 34616879 PMC8489945

[57] Schep AN, Wu B, Buenrostro JD, and Greenleaf WJ (2017). chrom-VAR: inferring transcription-factor-associated accessibility from single-cell epigenomic data. Nat. Methods 14, 975–978. 10.1038/nmeth.4401. 28825706 PMC5623146

[58] Lukhele S, Rabbo DA, Guo M, Shen J, Elsaesser HJ, Quevedo R, Carew M, Gadalla R, Snell LM, Mahesh L, (2022). The transcription factor IRF2 drives interferon-mediated CD8(+) T cell exhaustion to restrict anti-tumor immunity. Immunity 55, 2369–2385.e10. 10.1016/j.immuni.2022.10.020. 36370712 PMC9809269

[59] Chen Z, Arai E, Khan O, Zhang Z, Ngiow SF, He Y, Huang H, Manne S, Cao Z, Baxter AE, (2021). In vivo CD8(+) T cell CRISPR screening reveals control by Fli1 in infection and cancer. Cell 184, 1262–1280.e22. 10.1016/j.cell.2021.02.019. 33636129 PMC8054351

[60] Teijaro JR, Ng C, Lee AM, Sullivan BM, Sheehan KCF, Welch M, Schreiber RD, de la Torre JC, and Oldstone MBA (2013). Persistent LCMV infection is controlled by blockade of type I interferon signaling. Science 340, 207–211. 10.1126/science.1235214. 23580529 PMC3640797

[61] Wilson EB, Yamada DH, Elsaesser H, Herskovitz J, Deng J, Cheng G, Aronow BJ, Karp CL, and Brooks DG (2013). Blockade of chronic type I interferon signaling to control persistent LCMV infection. Science 340, 202–207. 10.1126/science.1235208. 23580528 PMC3704950

[62] Beltra J-C, Abdel-Hakeem MS, Manne S, Zhang Z, Huang H, Kurachi M, Su L, Picton L, Muroyama Y, Casella V, (2022). Enhanced STAT5a activation rewires exhausted CD8 T cells during chronic stimulation to acquire a hybrid durable effector like state. Preprint at bioRxiv. 10.1101/2022.10.03.509766.

[63] Liu J, Zhu S, Hu W, Zhao X, Shan Q, Peng W, and Xue HH (2023). CTCF mediates CD8+ effector differentiation through dynamic redistribution and genomic reorganization. J. Exp. Med 220, e20221288. 10.1084/jem.20221288. 36752796 PMC9948760

[64] Shan Q, Zhu S, Chen X, Liu J, Yuan S, Li X, Peng W, and Xue HH (2022). Tcf1-CTCF cooperativity shapes genomic architecture to promote CD8(+) T cell homeostasis. Nat. Immunol 23,1222–1235. 10.1038/s41590-022-01263-6. 35882936 PMC9579964

[65] Shan Q, Hu S, Chen X, Danahy DB, Badovinac VP, Zang C, and Xue HH (2021). Ectopic Tcf1 expression instills a stem-like program in exhausted CD8(+) T cells to enhance viral and tumor immunity. Cell. Mol. Immunol 18, 1262–1277. 10.1038/s41423-020-0436-5. 32341523 PMC8093427

[66] Behera V, Evans P, Face CJ, Hamagami N, Sankaranarayanan L, Keller CA, Giardine B, Tan K, Hardison RC, Shi J, and Blobel GA (2018). Exploiting genetic variation to uncover rules of transcription factor binding and chromatin accessibility. Nat. Commun 9, 782. 10.1038/s41467-018-03082-6. 29472540 PMC5823854

[67] Dubois-Chevalier J, Oger F, Dehondt H, Firmin FF, Gheeraert C, Staels B, Lefebvre P, and Eeckhoute J (2014). A dynamic CTCF chromatin binding landscape promotes DNA hydroxylmethylation and transcriptional induction of adipocyte differentiation. Nucleic Acids Res. 42, 10943–10959. 10.1093/nar/gku780. 25183525 PMC4176165

[68] Wang H, Maurano MT, Qu H, Varley KE, Gertz J, Pauli F, Lee K, Canfield T, Weaver M, Sandstrom R, (2012). Widespread plasticity in CTCF occupancy linked to DNA methylation. Genome Res. 22, 1680–1688. 10.1101/gr.136101.111. 22955980 PMC3431485

[69] Qi Q, Cheng L, Tang X, He Y, Li Y, Yee T, Shrestha D, Feng R, Xu P, Zhou X, (2021). Dynamic CTCF binding directly mediates interactions among cis-regulatory elements essential for hematopoiesis. Blood 137, 1327–1339. 10.1182/blood.2020005780. 33512425 PMC7955410

[70] Marino MM, Rega C, Russo R, Valletta M, Gentile MT, Esposito S, Baglivo I, De Feis I, Angelini C, Xiao T, (2019). Interactome mapping defines BRG1, a component of the SWI/SNF chromatin remodeling complex, as a new partner of the transcriptional regulator CTCF. J. Biol. Chem 294, 861–873. 10.1074/jbc.RA118.004882. 30459231 PMC6341399

[71] Valletta M, Russo R, Baglivo I, Russo V, Ragucci S, Sandomenico A, Iaccarino E, Ruvo M, De Feis I, Angelini C, (2020). Exploring the Interaction between the SWI/SNF chromatin remodeling complex and the zinc finger factor CTCF. Int. J. Mol. Sci 21, 8950. 10.3390/ijms21238950. 33255744 PMC7728349

[72] Alpsoy A, Sood S, and Dykhuizen EC (2021). At the Crossroad of gene regulation and genome organization: potential roles for ATP-dependent chromatin remodelers in the regulation of CTCF-mediated 3D architecture. Biology 10, 272. 10.3390/biology10040272. 33801596 PMC8066914

[73] Di Pilato M, Kfuri-Rubens R, Pruessmann JN, Ozga AJ, Messemaker M, Cadilha BL, Sivakumar R, Cianciaruso C, Warner RD, Marangoni F, (2021). CXCR6 positions cytotoxic T cells to receive critical survival signals in the tumor microenvironment. Cell 184, 4512–4530.e22. 10.1016/j.cell.2021.07.015. 34343496 PMC8719451

[74] Wang B, Wang Y, Sun X, Deng G, Huang W, Wu X, Gu Y, Tian Z, Fan Z, Xu Q, (2021). CXCR6 is required for antitumor efficacy of intratumoral CD8(+) T cell. J. Immunother. Cancer 9, e003100. 10.1136/jitc-2021-003100. 34462326 PMC8407215

[75] Bluemn T, Schmitz J, Zheng Y, Burns R, Zheng S, DeJong J, Christiansen L, Arnold O, Izaguirre-Carbonell J, Wang D, (2022). Differential roles of BAF and PBAF subunits, Arid1b and Arid2, in MLL-AF9 leukemogenesis. Leukemia 36, 946–955. 10.1038/s41375-021-01505-w. 35022500 PMC10095935

[76] Prévost-Blondel A, Zimmermann C, Stemmer C, Kulmburg P, Rosenthal FM, and Pircher H (1998). Tumor-infiltrating lymphocytes exhibiting high ex vivo cytolytic activity fail to prevent murine melanoma tumor growth in vivo. Journal of immunology 161, 2187–2194. 9725210

[77] Butler A, Hoffman P, Smibert P, Papalexi E, and Satija R (2018). Integrating single-cell transcriptomic data across different conditions, technologies, and species. Nat. Biotechnol 36, 411–420. 10.1038/nbt.4096. 29608179 PMC6700744

[78] Stuart T, Butler A, Hoffman P, Hafemeister C, Papalexi E, Mauck WM 3rd, Hao Y, Stoeckius M, Smibert P, and Satija R (2019). Comprehensive integration of single-cell data. Cell 177, 1888–1902.e21. 10.1016/j.cell.2019.05.031. 31178118 PMC6687398

[79] Hao Y, Hao S, Andersen-Nissen E, Mauck WM 3rd, Zheng S, Butler A, Lee MJ, Wilk AJ, Darby C, Zager M, (2021). Integrated analysis of multimodal single-cell data. Cell 184, 3573–3587.e29. 10.1016/j.cell.2021.04.048. 34062119 PMC8238499

[80] Wickham H, Averick M, Bryan J, Chang W, D’Agostino McGowan L, François R, Grolemund G, Hayes A, Henry L, Hester J, (2019). Welcome to the Tidyverse. Journal of Open Source Software 4, 1686. 10.21105/joss.01686.

[81] Gu Z, Eils R, and Schlesner M (2016). Complex heatmaps reveal patterns and correlations in multidimensional genomic data. Bioinformatics 32, 2847–2849. 10.1093/bioinformatics/btw313. 27207943

[82] Picelli S, Faridani OR, Björklund AK, Winberg G, Sagasser S, and Sandberg R (2014). Full-length RNA-seq from single cells using Smartseq2. Nat. Protoc 9, 171–181. 10.1038/nprot.2014.006. 24385147

[83] Ewels PA, Peltzer A, Fillinger S, Patel H, Alneberg J, Wilm A, Garcia MU, Di Tommaso P, and Nahnsen S (2020). The nf-core framework for community-curated bioinformatics pipelines. Nat. Biotechnol 38, 276–278. 10.1038/s41587-020-0439-x. 32055031

[84] Patro R, Duggal G, Love MI, Irizarry RA, and Kingsford C (2017). Salmon provides fast and bias-aware quantification of transcript expression. Nat. Methods 14, 417–419. 10.1038/nmeth.4197. 28263959 PMC5600148

[85] Wu T, Hu E, Xu S, Chen M, Guo P, Dai Z, Feng T, Zhou L, Tang W, Zhan L, (2021). clusterProfiler 4.0: a universal enrichment tool for interpreting omics data. Innovation 2, 100141. 10.1016/j.xinn.2021.100141. 34557778 PMC8454663

[86] Subramanian A, Tamayo P, Mootha VK, Mukherjee S, Ebert BL, Gillette MA, Paulovich A, Pomeroy SL, Golub TR, Lander ES, and Mesirov JP (2005). Gene set enrichment analysis: a knowledge-based approach for interpreting genome-wide expression profiles. Proc. Natl. Acad. Sci. USA 102, 15545–15550. 10.1073/pnas.0506580102. 16199517 PMC1239896

[87] Liberzon A, Birger C, Thorvaldsdóttir H, Ghandi M, Mesirov JP, and Tamayo P (2015). The Molecular Signatures Database (MSigDB) hallmark gene set collection. Cell Syst. 1, 417–425. 10.1016/j.cels.2015.12.004. 26771021 PMC4707969

[88] Corces MR, Trevino AE, Hamilton EG, Greenside PG, Sinnott-Armstrong NA, Vesuna S, Satpathy AT, Rubin AJ, Montine KS, Wu B, (2017). An improved ATAC-seq protocol reduces background and enables interrogation of frozen tissues. Nat. Methods 14, 959–962. 10.1038/nmeth.4396. 28846090 PMC5623106

[89] Li H, and Durbin R (2009). Fast and accurate short read alignment with Burrows-Wheeler transform. Bioinformatics 25, 1754–1760. 10.1093/bioinformatics/btp324. 19451168 PMC2705234

[90] Zhang Y, Liu T, Meyer CA, Eeckhoute J, Johnson DS, Bernstein BE, Nusbaum C, Myers RM, Brown M, Li W, and Liu XS (2008). Model-based analysis of ChIP-seq (MACS). Genome Biol. 9, R137. 10.1186/gb-2008-9-9-r137. 18798982 PMC2592715

[91] Love MI, Huber W, and Anders S (2014). Moderated estimation of fold change and dispersion for RNA-seq data with DESeq2. Genome Biol. 15, 550. 10.1186/s13059-014-0550-8. 25516281 PMC4302049

[92] Ross-Innes CS, Stark R, Teschendorff AE, Holmes KA, Ali HR, Dunning MJ, Brown GD, Gojis O, Ellis IO, Green AR, (2012). Differential oestrogen receptor binding is associated with clinical outcome in breast cancer. Nature 481, 389–393. 10.1038/nature10730. 22217937 PMC3272464

[93] Heinz S, Benner C, Spann N, Bertolino E, Lin YC, Laslo P, Cheng JX, Murre C, Singh H, and Glass CK (2010). Simple combinations of lineage-determining transcription factors prime cis-regulatory elements required for macrophage and B cell identities. Mol. Cell 38, 576–589. 10.1016/j.molcel.2010.05.004. 20513432 PMC2898526

[94] Thorvaldsdóttir H, Robinson JT, and Mesirov JP (2013). Integrative Genomics Viewer (IGV): high-performance genomics data visualization and exploration. Brief. Bioinform 14, 178–192. 10.1093/bib/bbs017. 22517427 PMC3603213

[95] Robinson JT, Thorvaldsdóttir H, Winckler W, Guttman M, Lander ES, Getz G, and Mesirov JP (2011). Integrative genomics viewer. Nat. Biotechnol 29, 24–26. 10.1038/nbt.1754. 21221095 PMC3346182

[96] Stuart T, Srivastava A, Madad S, Lareau CA, and Satija R (2021). Single-cell chromatin state analysis with Signac. Nat. Methods 18, 1333–1341. 10.1038/s41592-021-01282-5. 34725479 PMC9255697

[97] Castro-Mondragon JA, Riudavets-Puig R, Rauluseviciute I, Lemma RB, Turchi L, Blanc-Mathieu R, Lucas J, Boddie P, Khan A, Manosalva Pérez N, (2022). Jaspar 2022: the 9th release of the open-access database of transcription factor binding profiles. Nucleic Acids Res. 50, D165–D173. 10.1093/nar/gkab1113. 34850907 PMC8728201

