# Supplementary material for: Loss of PBAF promotes expansion and effector differentiation of CD8+ T cells during chronic viral infection and cancer
Source: Cell Rep. Author manuscript; Available in PMC 2023 Oct 23. (PMC10592487; doi:10.1016/j.celrep.2023.112649)

Cell Reports, Volume *42*

Supplemental information

Loss of PBAF promotes expansion and effector differentiation of CD8^{+} T cells during chronic viral infection and cancer

Arjun Kharel, Jian Shen, Ryan Brown, Yao Chen, Christine Nguyen, Donia Alson, Theresa Bluemn, Jie Fan, Kexin Gai, Bin Zhang, Matthew Kudek, Nan Zhu, and Weiguo Cui

Supplemental Information

Figure S1. Related to Figure 1. Hematopoietic loss of Arid2 promotes $CD8^+$ T cell expansion and $Gp_{33\text{-}41}^+$ peptide specific $CX3CR1^+$ $CD8^+$ T cell differentiation.
(A-D) Representative flow plots and summary data showing the proportion, subset distribution, and total number of $GP33^+$ splenic CD8 T cells. (E-G) Summary bar plot displaying the frequency of major lymphoid and myeloid populations.
Summary data (mean ± SEM) are pooled from with 1 experiment with at 3 mice/group per experiment. Data are representative of one experiment. $^*p < 0.05$, $^{**}p < 0.01$, $^{***}p < 0.0001$.

Figure S2. Related to Figure 1. Verification of mixed bone marrow chimera reconstitution and subset specific expression of key transcription factors.
(A-C) Flow plots showing the 70:30 reconstitution of wild-type and $Arid2^{-/-}$ CD8 T cells in mixed bone marrow chimera model. (D-E) Summary data and Representative flow plots showing per cell expression (gMFI) of TCF-1 and TOX in the three major subsets from splenic $CD44^+PD\text{-}1^+CD8^+$ T cells. Summary data (mean ± SEM) are pooled from 1 experiment with at least 4 mice/group per experiment. Data are representative of one independent experiments. $^*p < 0.05$, $^{**}p < 0.01$, $^{***}p < 0.0001$.

Figure S3. Related to Figure 2 and Figure 3. Verification of Arid2 and Pbrm1 CRISPR-cas9 deletion, endogenous and P14 $CD8^+$ T cell responses at day 21 p.i.
(A-C) TIDE assay on $CD8^+$ T cells transduced with sgCtrl, *Arid2* gRNA and *Pbrm1* gRNA. (D) Western blot analysis of PBRM1 expression in CD8 T cells transduced with scrambled (ctrl) gRNA and *Pbrm1* gRNA. GAPDH antibody used as loading control. (E) Representative flow plot showing the proportion and subset distribution of $GP276^+$ endogenous $CD8^+$ T cells at day 21 p.i. (F) Heatmap of interferon pathway related genes with differential expression between control and Arid2-deficient conditions. (G-H) Summary data showing frequency of IFNγ expressing cells and gMFI of Granzyme B of sgCtrl, *Arid2*-deleted and *Pbrm1*-deleted $CD8^+$ T cells at day 21 p.i. TIDE assay data are representative of two independent experiments. Representative flow plots are representative of one experiment. Summary data (mean ± SEM) for IFNγ and gMFI are pooled from 2 experiments with at 4 mice/group per experiment. Data are representative of three experiments. $^*p < 0.05$, $^{**}p < 0.01$, $^{***}p < 0.0001$.

Figure S4. Related to Figure 5. Single cell multiomics reveals PBAF-regulated exhaustion and proliferation programs in a subset specific manner (A) wnnUMAP of sgCtrl, sgArid2, and sgPbrm1 (B-C) Dot plot showing expression of cluster markers. Dot size denotes the number of cells with a particular gene expressed, intensity of dot color indicates the expression level of RNA expression (B), Transcription Start Stie (TSS) Accessibility (D) Module scores of the top 100 differentially expressed genes from previously identified progenitor, effector, and exhausted splenic $CD8^+$ T cells for sgCtrl, sgArid2, and sgPbrm1. (E) Coverage plot of the *Bik* locus showing ATAC-seq chromatin accessibility.

Figure S5. Related to Figure 6. Single cell multiomics reveals unique TF motif accessibility that regulates $CD8^+$ T cell differentiation.

(A) Dot plot of motif accessibility for each cluster. Dot size denotes the number of cells with a particular motif expressed, intensity of dot color indicates the ChromVar deviation score. (B) Volcano plot showing motifs that are differentially accessible between sgArid2 and sgCtrl in the progenitor cluster. (C) Volcano plot showing differentially accessible peaks that contain the CTCF motif between sgPbrm1 and sgCtrl. (D) Coverage plot of the *E2f2* locus with the CTCF motif location highlighted in grey (E)

Figure S6. Related to Figure 7. P14 CD^{8+} T cell responses following co-transfer of sgCtrl and *Arid2*-deleted $CD8^+$ T cells.

(A-B) Summary plot showing the number of $CD45.1^+$ $CD8^+$ T cells in the tumor and draining lymph nodes (C) Heatmap showing the mean expression of key markers between sgCtrl and *Arid2*-deleted $CD8^+$ T cells. (D-G) UMAP plot displaying three major clusters and their respective marker expressions. Data (Mean+/− S.E.M.) in (H) P14 $CD8^+$ T cells transduced with control and *Arid2* guide RNA were activated *in-vitro* for 3 days, mixed in 1:1 ratio and adoptively transferred into mice congenic C57BL/6 mice harboring B16-$GP_{33\text{-}41}$ tumor for 3 days. (I-K) Representative flow plot and summary data showing the frequency and number of sgCtrl and sgArid2 P14 $CD8^+$ T cells from tumor. (L-M) Representative flow plot and summary data showing the frequency of $PD\text{-}1^+TOX^+$ $CD8^+$ T cells in the two groups. (N) Summary data showing representative flow plot and per cell expression (gMFI) of Granzyme B of P14 $CD8^+$ T cells. (A, B) is pooled and are from at least 3 mice/group/experiment and are representative of at least 3 independent experiments. Data (Mean+/− S.E.M.) in (A) is from 3 mice/group and one experiment and (B) is pooled from 3 independent experiments with atleast 3 mice/group/experiment. UMAP is generated using Cytobank following concatenation of $CD45.1^+$

$CD8^+$ T cells: (n=6) sgCtrl transduced cell and (n=6) sg*Arid2* transduced cell. Heatmap were generated using hyperbolic arcsine (arcsinh) transformation. (H-N) is pooed from one independent experiment with 5 mice/group. *p<0.05 **p<0.01 ***p<0.000.

Supplemental Figure 1

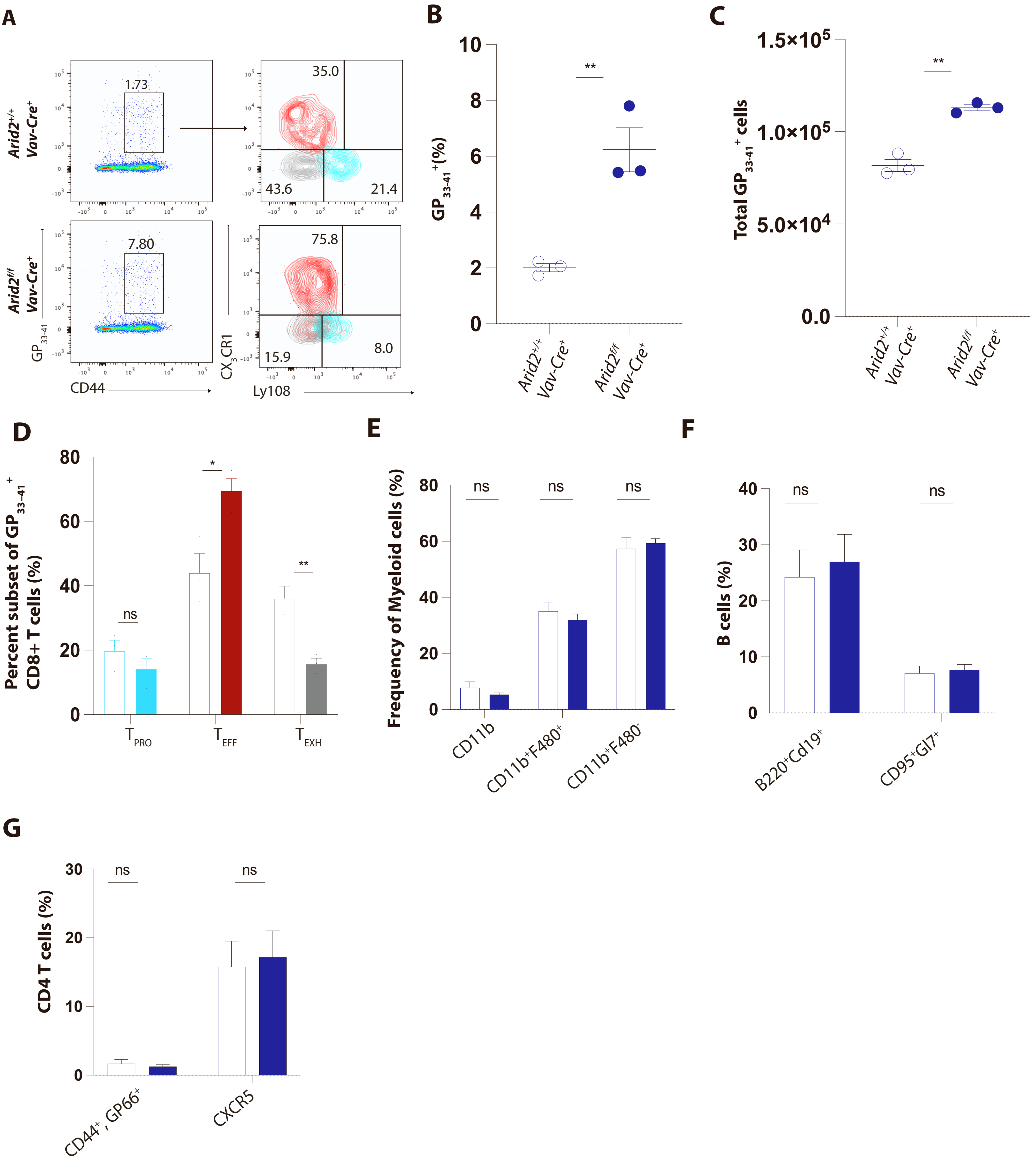

Supplemental Figure 2

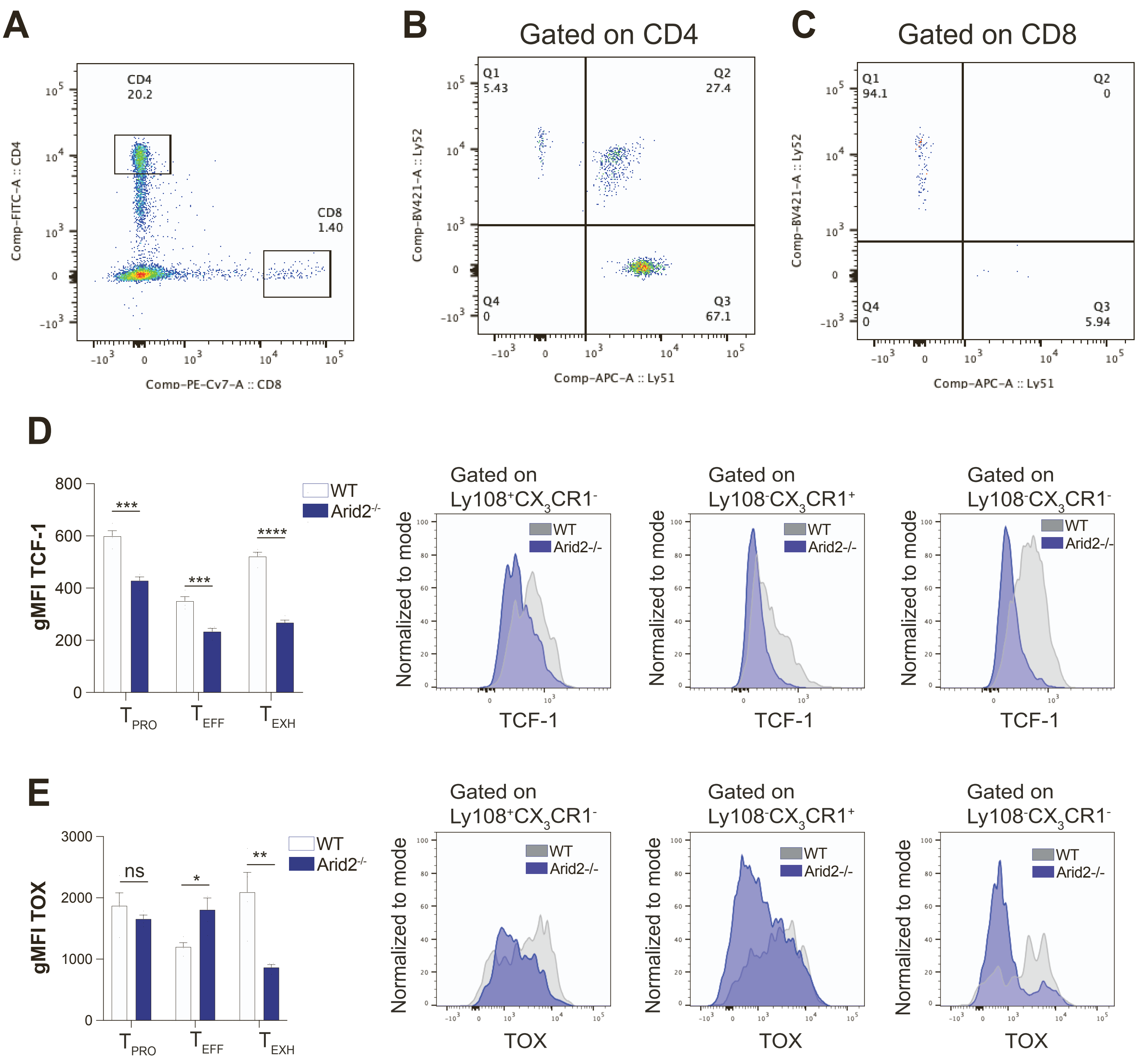

Supplemental Figure 3

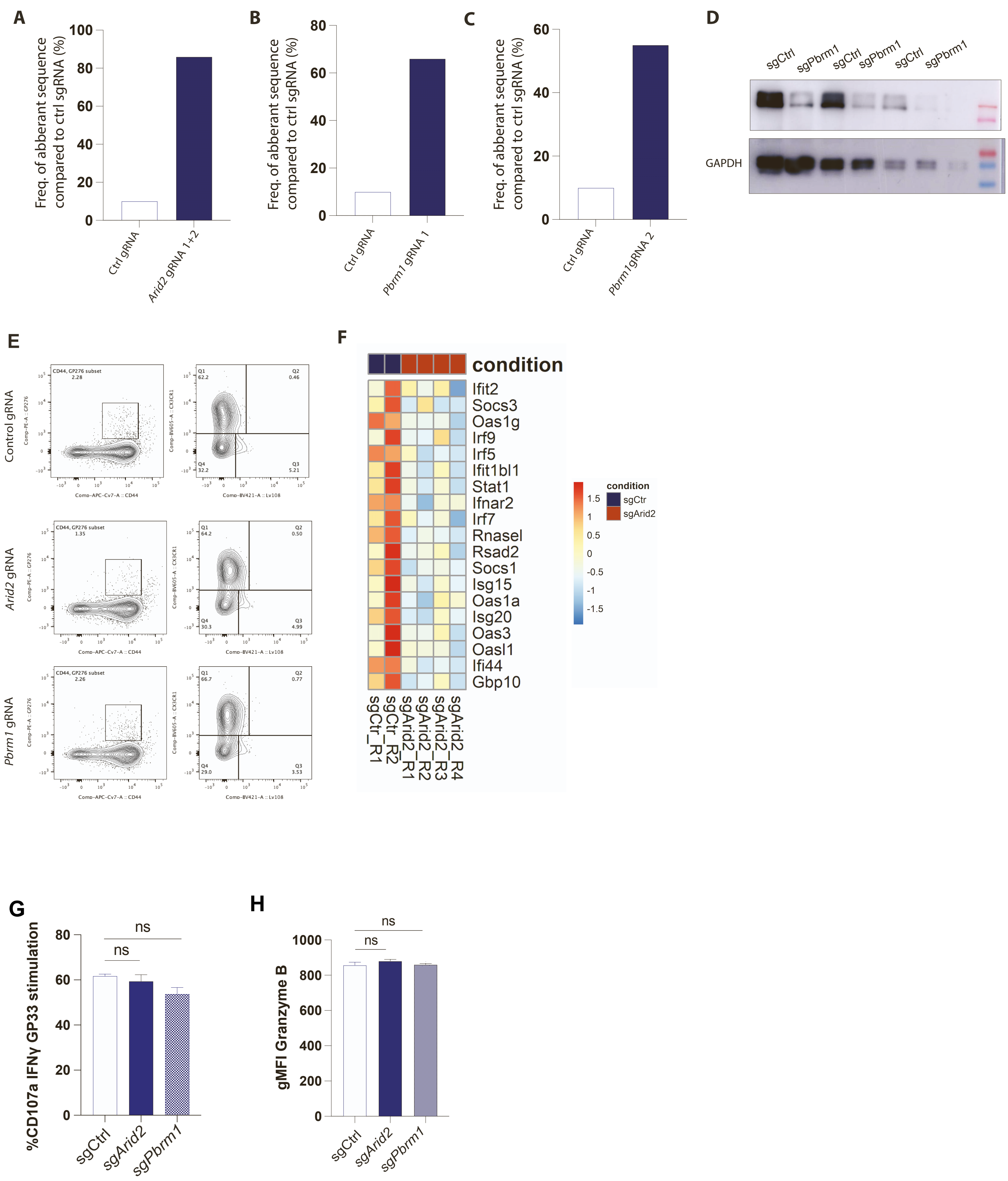

Supplemental Figure 4

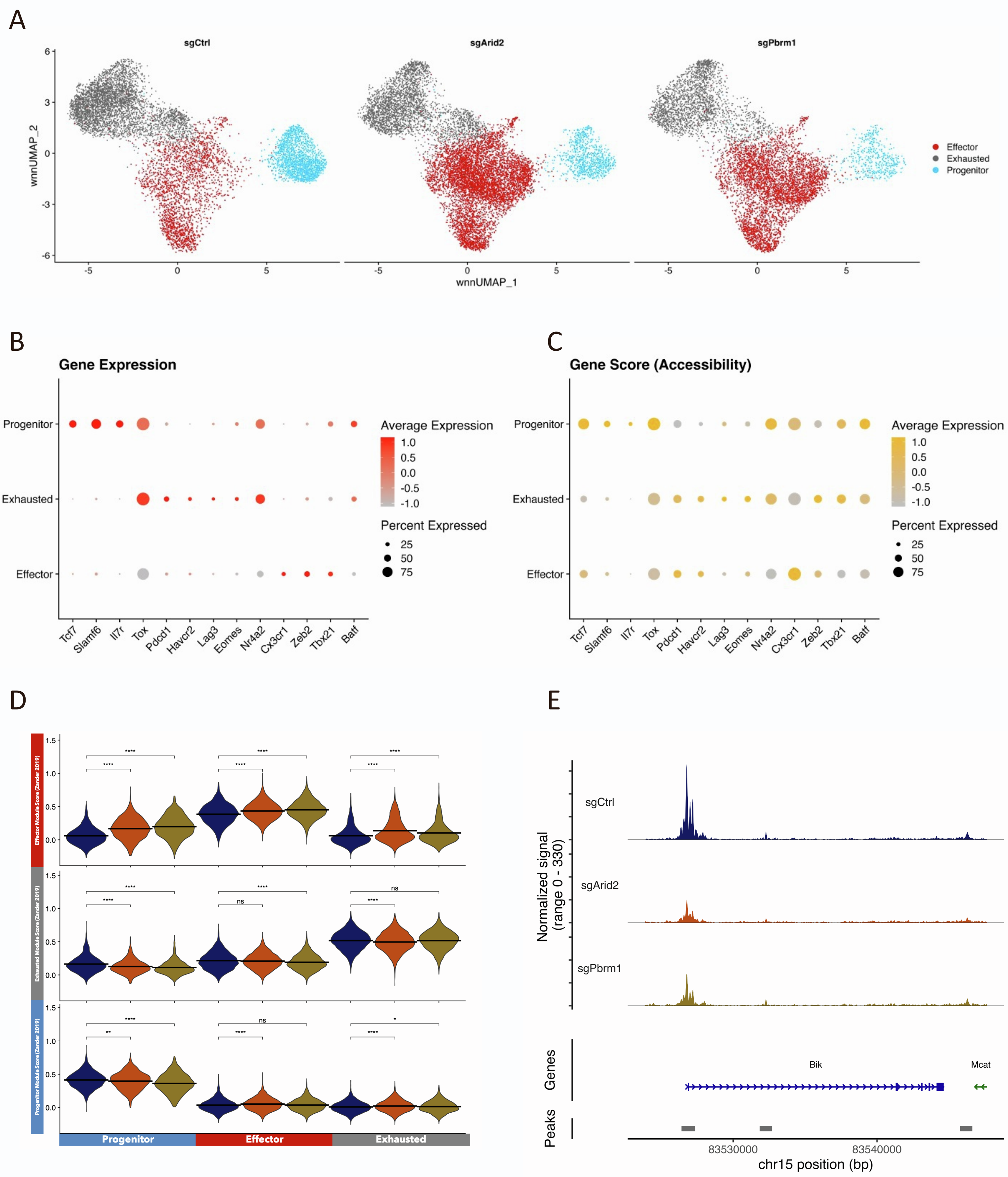

Supplemental Figure 5

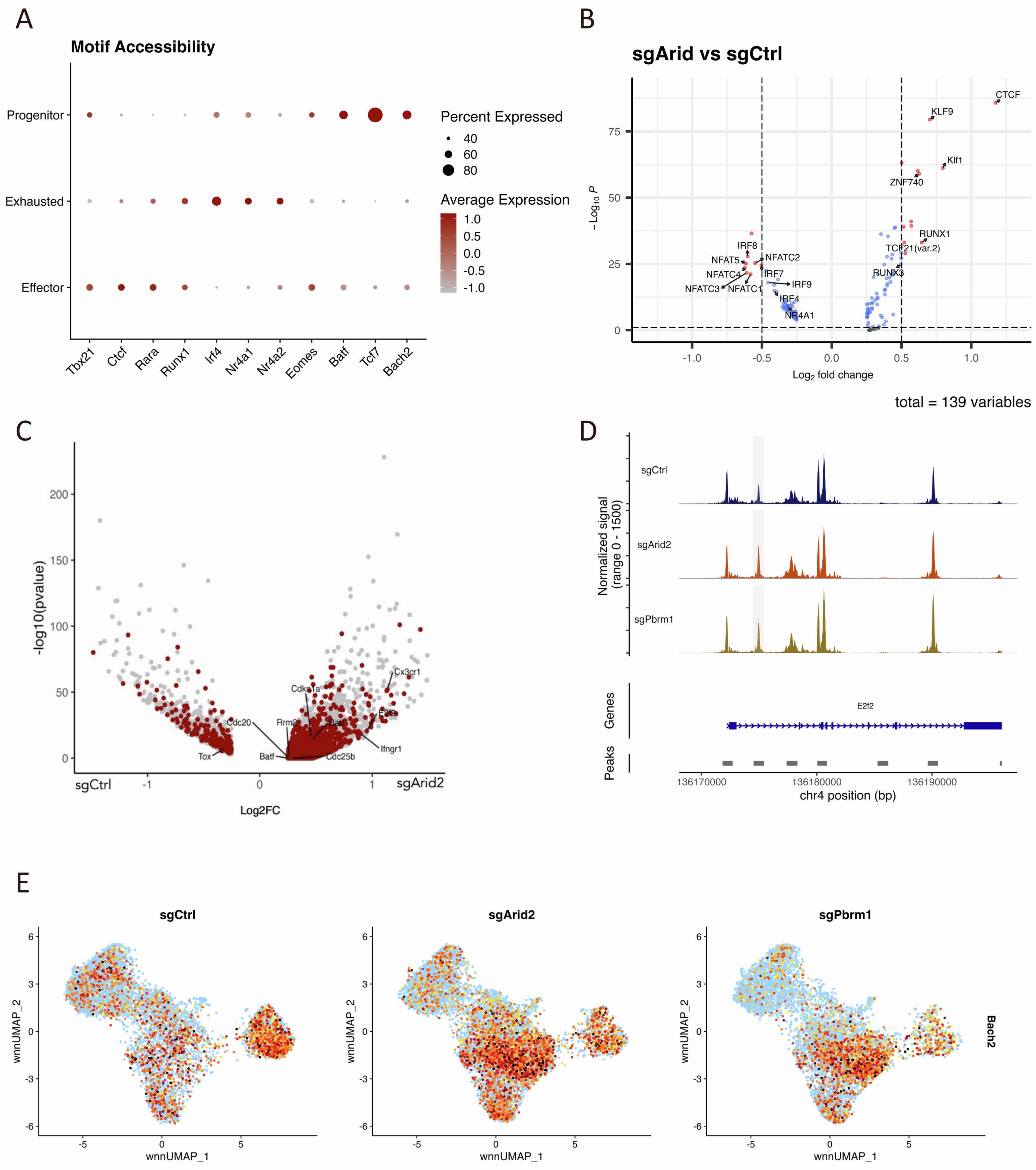

Supplemental Figure 6

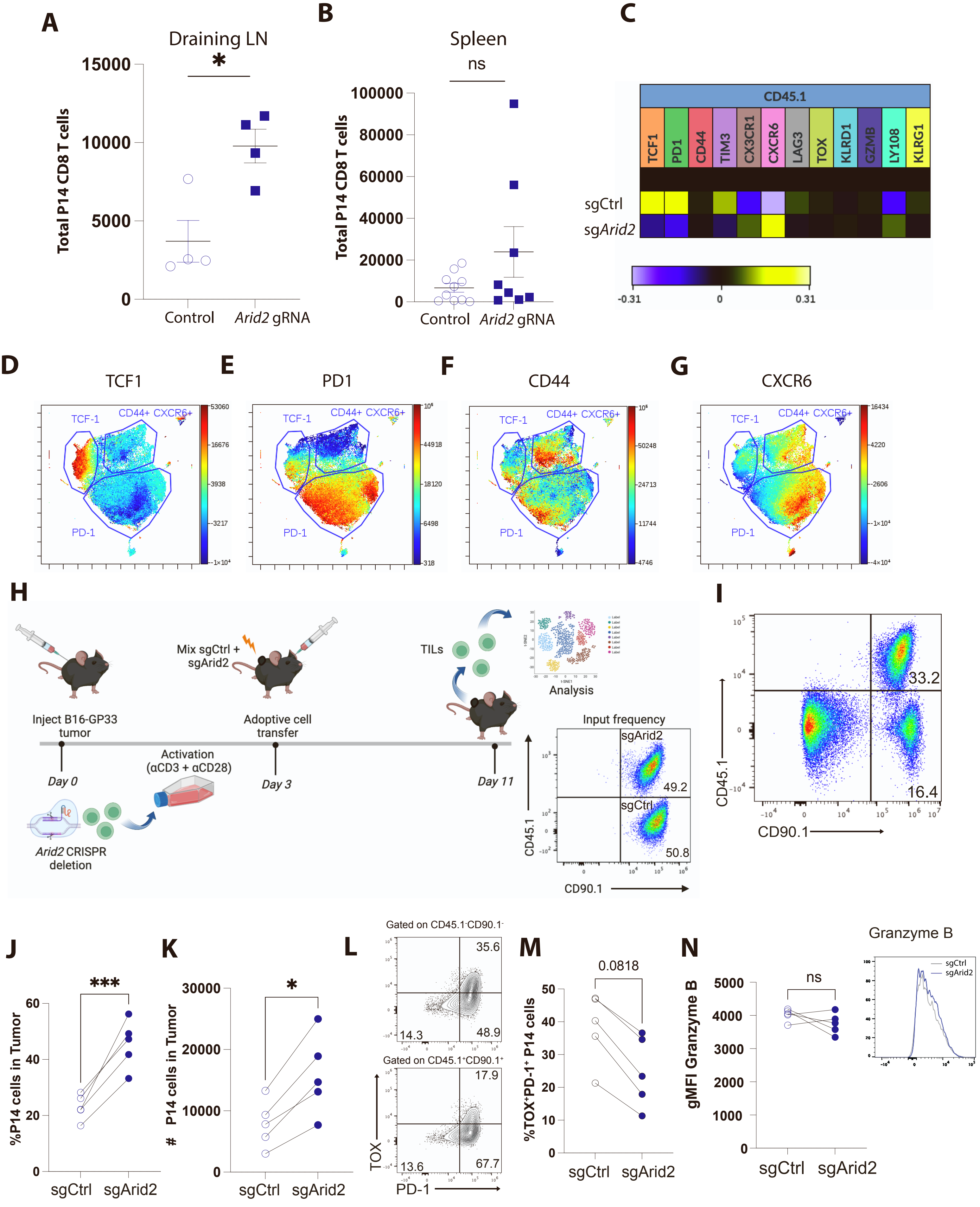

Supplement: 1 [file NIHMS1912757-supplement-1.pdf]
